# Pan-cancer analysis of the transcriptional expression of histone acetylation enzymes in solid tumors defines a new classification scheme for gliomas

**DOI:** 10.3389/fimmu.2024.1523034

**Published:** 2025-01-21

**Authors:** Junhao Zhang, Lingbo Li, Aiwei Tang, Chucheng Wang, Yupeng Wang, Yongqi Hu, Guangting He, Wangjun Liao, Rui Zhou

**Affiliations:** ^1^ Department of Oncology, Nanfang Hospital, Southern Medical University, Guangzhou, Guangdong, China; ^2^ Department of Anesthesiology, Nanfang Hospital, Southern Medical University, Guangzhou, Guangdong, China; ^3^ Guangdong Provincial Key Laboratory of Precision Anesthesia and Perioperative Organ Protection, Guangzhou, Guangdong, China; ^4^ Cancer Center, The Sixth Affiliated Hospital, School of Medicine, South China University of Technology, Foshan, China; ^5^ Foshan Key Laboratory of Translational Medicine in Cancer, The Sixth Affiliated Hospital, School of Medicine, South China University of Technology, Foshan, China

**Keywords:** histone acetylation, molecular classification, pan-cancer, glioma, prognosis

## Abstract

**Introduction:**

The altered expression of genes encoding histone acetyltransferases (HATs) and histone deacetylases (HDACs) has been implicated in the tumorigenesis and progression of various solid tumors. However, systematic characterization of the transcriptomic landscape and clinical relevance of HATs and HDACs in pan-cancer contexts remains lacking.

**Methods:**

Transcriptome and clinical data of 9,483 patients across 31 tumor types from The Cancer Genome Atlas were collected for systematic pan-cancer analysis. Additional glioma-specific datasets (Chinese Glioma Genome Atlas, GlioVis, GSE43378, and GSE182109) were also collected to validate the transcriptional characteristics of HATs and HDACs in gliomas. Consensus clustering analysis was applied to identify distinct expression patterns of HATs and HDACs.

**Results:**

Based on the transcriptomic data of 25 genes encoding 9 HATs and 16 HDACs, we identified five major subtypes across 31 cancer types (AC-I to AC-V). Notably, the AC-V subtype comprised over 95% of glioma patients, suggesting glioma patients exhibited distinct expression patterns of histone acetylation-modifying enzymes compared to patients with other solid tumors. Therefore, we re-conducted the consensus clustering analysis specifically within the context of gliomas and identified five subtypes, denoted “AC-GI” to “AC-GV”, which were characterized by differences in HATs/HDACs expression patterns, biological and immune status, genetic alterations, and clinical outcomes. The AC-GII patients exhibited the best prognosis and were sensitive to temozolomide, while AC-GV patients had the poorest prognosis and the lowest sensitivity to temozolomide among all subtypes. Moreover, based on the Connectivity Map database analysis and experimental verification, we identified several pan-HDAC inhibitors that could serve as sensitizers for temozolomide therapy in AC-GV patients, such as panobinostat and scriptaid. Considering the distinctive clinical characteristics of patients with AC-GII and AC-GV, we constructed the “ACG score” model capable of effectively recognizing patients with these subtypes and predicting patient prognosis.

**Conclusion:**

Herein, we established novel biologically and clinically relevant molecular classifications for pan-solid tumors and gliomas based on transcriptional expression profiles of HATs and HDACs. Moreover, the ACG score model, calculated by the transcriptional expression of 29 genes, was not only an independent prognostic factor for glioma patients, but can also provide valuable references for promoting more effective therapeutic strategies.

## 1 Introduction

As a primary epigenetic mechanism influencing oncogenesis, the acetylation of histone lysine residues, modulated by the opposing activities of histone acetyltransferases (HATs) and histone deacetylases (HDACs), can markedly affect chromatin compaction, further impacting transcription factors accessing the promoter region and the transcriptional expression of genes (1–4). Considering that HDACs are frequently dysregulated across various tumors and inhibiting their activity could effectively disrupt tumor cell viability, HDACs have emerged as promising therapeutic targets for multiple tumor types (5, 6). However, although *in vitro* and *in vivo* studies have shown promising results (7, 8), clinical trials for HDAC inhibitors (HDACis) in solid tumors have been unsatisfactory, possibly owing to the lack of patient stratification, with existing inhibitors targeting multiple HDACs, potentially eliciting unwanted side effects (9–11). Therefore, elucidating the expression features of histone acetylation-modifying enzymes at the pan-cancer level could provide a more comprehensive view of the acetylation-deacetylation balance, which may help unravel both the commonalities and heterogeneity of various human malignancies, offering insights for screening patients suitable for HDACis therapy.

Recently, the mRNA expression of histone acetylation-modifying enzymes has been used to define molecular subtypes with substantial prognostic differences in patients with gastric and colon cancer (12, 13). However, systematic efforts to characterize the transcriptomic landscape and clinical relevance of HATs and HDACs in pan-cancer backgrounds are lacking. In this study, we first conducted a pan-cancer analysis of the transcriptomic expression of 9 HATs and 16 HDACs to provide a preliminary overview of mRNA expression patterns of histone acetylation-modifying enzymes across various tumor types. More importantly, given our findings suggested that glioma displayed unique expression patterns of HATs and HDACs compared to other solid tumors, we specifically performed consensus clustering analysis on specimens of patients with glioma to identify molecular subtypes with distinct biological features, tumor microenvironment (TME) and clinical outcome.

## 2 Materials and methods

### 2.1 Data retrieval

The cancer-associated datasets, including mRNA sequencing (n = 10,079), clinical (n = 9,483), single nucleotide variation (SNV) (n = 10,234), copy number variation (CNV) (n = 10,878), microRNA data (n=10,818), and proteomic data (n=8,657) were gathered from the “TCGA pan-cancer” section of University of California Santa Cruz (UCSC) Xena database (https://xenabrowser.net). A total of 31 types of solid tumors were included in our pan cancer analysis (**Supplementary Table S1**). Particularly, we also collected the transcriptomic data of gliomas in Chinese Glioma Genome Atlas (CGGA, http://www.cgga.org.cn/), GlioVis (http://gliovis.bioinfo.cnio.es/), and GEO (http://www.ncbi.nlm.nih.gov/geo, GSE43378 and GSE182109) databases for exploring and validating our findings in glioma (**Supplementary Table S2**).

### 2.2 Consensus clustering analysis based on transcriptomic profiles of HATs and HDACs

We conducted an unsupervised clustering (K-means) analysis based on transcriptomic profiles of HATs and HDACs to delineate different acetylation regulator modification patterns. ConsensusClusterPlus (14) was applied to choose the optimal clustering number and assess clustering stability. To ensure both robustness and computational feasibility of the results, the parameters were chosen as follows: maxK (the maximum number of clusters) = 10, reps (the number of resampling iterations) = 1,000, pItem (the proportion of data points randomly sampled for each iteration) = 0.95, pFeature (the proportion of features considered in each iteration) = 1.

### 2.3 Prediction of molecular subtype by a combination of prediction analysis for microarray and nearest template prediction algorithms

To map the classification obtained from consensus clustering analysis on independent datasets, we first employed the prediction of molecular subtype by a combination of prediction analysis for microarray (PAM) algorithm to identify gene markers whose expression best discriminates each subtype based on the whole transcriptome (15). Then, the filtered gene sets were subsequently used as template features for nearest template prediction (NTP) (16), which is a correlation-based method specifically constructed to provide robust class predictions for high-dimensional and noisy gene expression data (17).

### 2.4 Biological process, metabolic activation and immune infiltration analysis

To analyze biological processes, we implemented gene set variation analysis (GSVA) (18) against a collection of 50 hallmark biological pathways and 186 Kyoto Encyclopedia of Genes and Genomes (KEGG) pathways as previously described (19, 20). The activation of metabolic gene signatures and immune cell signatures in each sample were analyzed using the “IOBR” R package (21). A total of 6 algorithms for estimating immune cell infiltration including quantTIseq, TIMER, EPIC, MCP-counter, ESTIMATE, and xCell were embedded in “IOBR”.

### 2.5 Trajectory analysis

The pseudotime analysis was conducted using the ‘monocle’ R package (22). Firstly, a monocle object was established utilizing the ‘newCellDataSet’ function. Following this, genes with a minimum expression level of 0.1 and expressed in at least 0.5% of samples were selected. Moreover, the ‘differentialGeneTest’ function was utilized for identifying genes for trajectory sequencing. Next, the ‘reduceDimension’ function was adopted for dimension reduction, applying the parameter ‘reduction_method = DDRTree’. Lastly, the samples were organized in a pseudotime sequence using the ‘orderCells’ function and visualization was implemented by the ‘plot_cell_trajectory’ function.

### 2.6 Somatic genetic mutation and miRNA enrichment analysis

For somatic genetic mutation analysis, the genetic mutation file was acquired using the TCGAbiolinks package (23), and the identification of significant cancer mutated genes (SMGs) was performed through the application of MutSigCV algorithm (24). Subsequently, we selected the top 100 SMGs based on their total mutation frequency and conducted a chi-square test to analyze the distribution of effective mutations among different groups as previously described (25, 26). As for the miRNA enrichment analysis, the pathway enrichment analysis based on miRNAs was conducted by using the miRNA Enrichment Analysis and Annotation Tool (https://ccb-compute2.cs.uni-saarland.de/mieaa2/).

### 2.7 Identify potential therapeutic drugs by Cancer Therapeutics Response Portal database analysis and Connectivity Map analysis

To identify potential therapeutic drugs for each subtype, we utilized pRRophetic package (27), which employs a ridge regression model based on the Cancer Therapeutics Response Portal (CTRP) database, to predict the single agent drug sensitivity of each sample. Drug sensitivity quantification was determined based on the area under the dose-response curve (AUC), where higher AUC values corresponded to diminished sensitivity. Moreover, to discover compounds that may improve chemosensitivity in subtypes resistant to chemotherapy, we performed Connectivity Map (CMap) analysis utilizing the online tool (https://clue.io/). We selected 300 differentially expressed genes (DEGs) with the most significant fold changes between samples in the targeted subtype and those in the control group (150 upregulated and 150 downregulated). These DEGs were input into the CMap database according to the website’s instructions. Compounds with enrichment scores below −90 were identified as potentially effective drugs.

### 2.8 *In vitro* and *in vivo* drug sensitivity assays

GBM cell line U87 was routinely maintained in Dulbecco’s Modified Eagle Medium (DMEM) supplemented with 10% fetal bovine serum at 37 °C with 5% CO2. U87 cell line was authenticated by the short tandem repeat profiling. As for the *in vitro* experiments, we measured cell survival of U87 cells under different treatment groups using CCK8, flow cytometry, and clonogenic assays, as previously described (28, 29). As for *in vivo* experiments, all animal experiments received approval from the Nanfang Hospital Animal Care and Use Committee and were conducted in accordance with the National Guidelines for Animal Experimentation. Female BALB/C nude mice, aged six weeks, were housed in a pathogen-free facility with unrestricted access to food and water, and were maintained under a 12-hour alternating dark/light cycle. The BALB/C nude mice received intracranial injections of 1 × 10^6^ cells, and tumor nodule volumes were monitored every other day. In the drug treatment experiments, mice were allocated to various treatment groups in a non-blinded manner once the tumor volume reached approximately 50 mm³. On the 14^th^ day, the mice were executed, and the transplanted tumors were removed for photography and weighing. The weight of transplanted tumors at the time of executing mice was used as an indicator to compare the efficacy of different drug treatment groups.

### 2.9 Identification of marker genes used for establishing signature scores

First, the TCGA/CGGA cohort was divided into the training set and validation set according to the histological types by utilizing the caret package (30). Then, we screened the candidate genes used for establishing signature scores through the following methodology: First, the limma package (31) was employed to identify DEGs between samples in the targeted subtype and samples in the control group. Only genes with |log_2_FC|>1.5 and adj p value < 0.05 were included in subsequent analysis. Afterwards, leave-one-out cross-validation (LOOCV) framework, a specialized form of cross-validation with each individual data point in the dataset is used as a test set and the remaining data points serve as the training set over each iteration, was employed to fit 105 prediction models generated by 11 independent methods, including Boruta feature selection (32), Random Survival Forest (33), Elastic Net, Generalized Boosted Regression Modeling (GBRM), Stepwise Cox, CoxBoost, Partial Least Squares Regression for Cox (34), Supervised Principal Components (35), Survival Support Vector Machine, Ridge, and Lasso (36), which were adopted to reduce the dimensions of DEGs and select genes with high C-index in both training and test cohort.

### 2.10 Single-cell RNA sequencing data processing

The glioblastoma multiforme (GBM) single-cell RNA sequencing (scRNA-seq) dataset GSE182109 (37) was analyzed utilizing the workflow of Seurat package (38). The unqualified cells were filtered out according to the following metrics: cells 1) expressing less than 300 genes; 2) with UMI counts < 500 or >200,000; 3) percentage of mitochondrial genes >20%; 4) ribosomal transcripts > 50% were filtered out. Genes expressed in fewer than three cells were also removed. Potential doublets or multiplets were identified and meticulously removed using Scrublet (39). After removing the low-quality and doublet cells, library size normalization was performed by adopting the LogNormalize method. Dimensionality reduction was subsequently conducted through principal component analysis (PCA), utilizing the matrix of the top 2,000 highly variable genes generated by the FindVariableFeatures function. We utilized Harmony (40) to correct batch effects. Then, the FindNeighbors and FindClusters functions were used to construct the shared nearest neighbors and major cell clusters, respectively. Dimensionality reduction and visualization of the clusters were conducted using Uniform Manifold Approximation and Projection. All cluster assignments underwent manual verification to ensure the precise partitioning of cells.

### 2.11 Statistical analysis

For the comparison between two groups of continuous variables, the Student’s t-test was employed to assess the disparities in parametrically distributed variables, while the Mann-Whitney U test was used to evaluate the nonparametrically distributed variables. When comparing more than two groups, we utilized the Kruskal-Wallis and one-way ANOVA tests for parametric and nonparametric variables, respectively. Analysis of categorical variables involved the use of chi-square and Fisher’s exact tests. Survival analyses were conducted utilizing Kaplan-Meier analysis, supplemented by the log-rank test as needed. The optimal threshold values for high- and low-score groups were determined utilizing the Survminer package. Statistical significance was established with a two-tailed p-value < 0.05. Version 4.3.3 of the R software was applied to conduct all the statistical analyses.

## 3 Results

### 3.1 Expression profiles of 25 genes encoding 9 HATs and 16 HDACs across 31 cancer types

**Figure 1:**
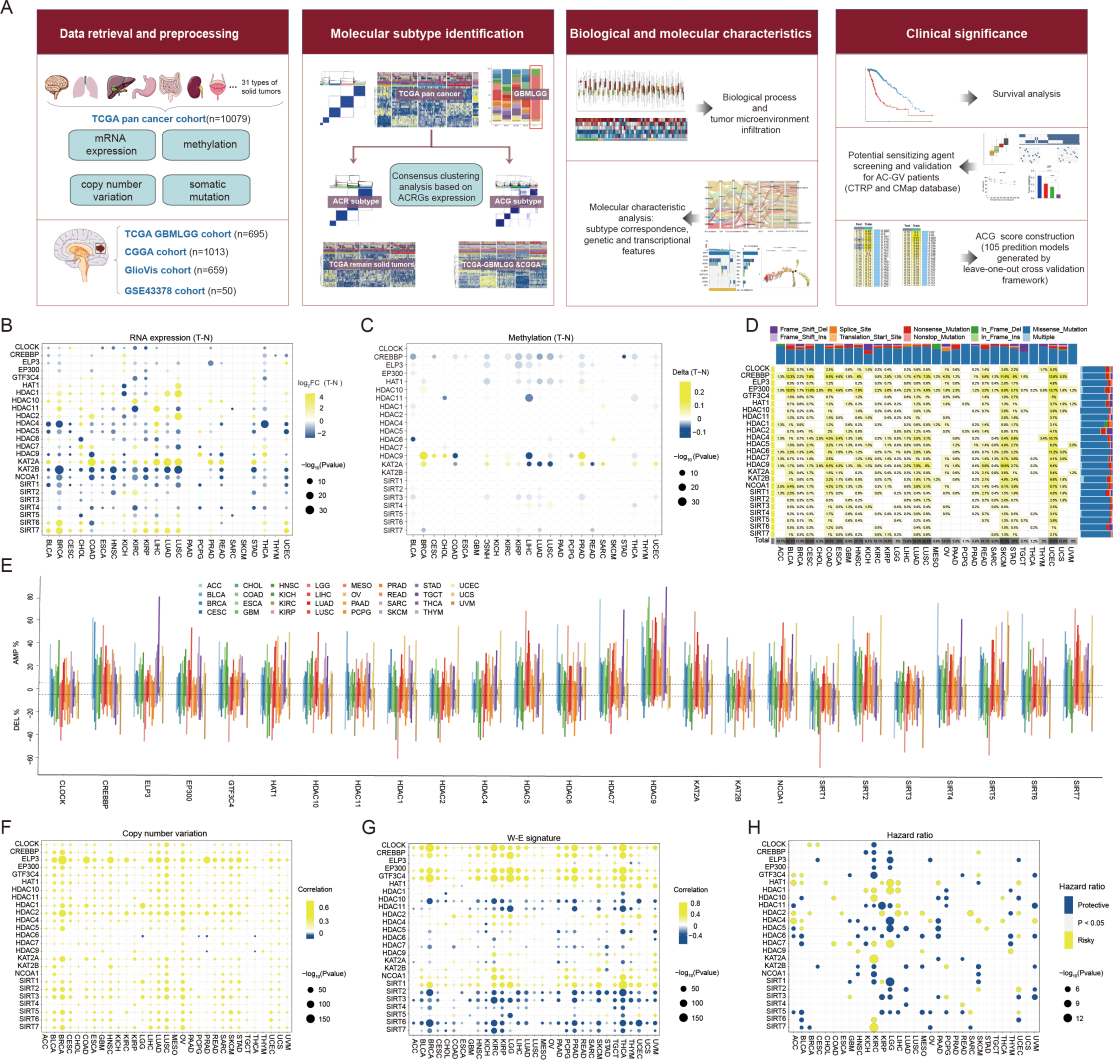
Landscape of histone acetylation regulator genes (ACRGs) in each cancer type of TCGA pan-cancer cohort. **(A)** Workflow of this study. **(B, C)** Dot plot depicted the differential mRNA expression level **(B)** and methylation level **(C)** of ACRGs between tumor and normal samples in each cancer type. The yellow dots indicated high gene expression or high methylation level in tumors and the blue dots represented low gene expression or low methylation level in tumors. **(D)** The mutation rate of each ACRG. The upper and right bar plot indicates the proportion of each variant type in different cancer types and ACRGs. **(E)** Histogram showed the frequency of somatic copy number alterations for each ACRG in each cancer type. **(F)** Dot plot depicted the correlation between somatic copy number alterations and mRNA expression of each ACRG in each cancer type. The yellow dots indicated positive correlations and the blue dots represented negative correlations. **(G)** Dot plot depicted the correlation between the W-E signature level and mRNA expression of each ACRG in each cancer type. The yellow dots indicated positive correlations and the blue dots represented negative correlations. **(H)** Dot plot depicted the correlation between overall survival (OS) and mRNA expression of ACRGs. The yellow and blue dots represented longer and shorter OS in the groups with higher mRNA levels of ACRGs, respectively.

**Figure 1A** illustrates the study workflow. We retrieved two reviews on histone acetylation modification (1, 41), and 29 recognized histone acetylation modification regulators were initially collected. After eliminating four HATs/HDACs genes that were absent from TCGA-pan cancer cohort, a total of 25 genes encoding 9 HATs and 16 HDACs, were collected and defined as histone acetylation regulator genes (ACRGs) for subsequent analysis. Comparing expression levels of these ACRGs in tumor and adjacent normal tissues, we found most ACRGs consistently dysregulated across various tumors. Specifically, HDAC4, HDAC5, KAT2B, NCOA1, SIRT1, and SIRT4 had lower expression in tumor tissues across 14, 12, 18, 17, 16, and 12 cancer types, respectively. Conversely, HDAC1, HDAC10, KAT2A, SIRT6, and SIRT7 were upregulated in 13, 16, 17, 14, and 12 cancer types (**Figure 1B**). Although transcriptomic levels varied significantly between tumor and normal tissues, methylation levels of most ACRGs did not, except for CREBBP, which was consistently hypomethylated across 15 tumor types (**Figure 1C**). Regarding somatic mutations, out of 9,477 samples, 1,796 (18.95%) had at least one ACRG mutation (defined as the total mutation rate), predominantly in CREBBP, EP300, and HDAC9. The total mutation rate was the highest in skin cutaneous melanoma (47.54%), followed by bladder urothelial carcinoma (40.15%) and uterine corpus endometrial carcinoma (35.59%) (**Figure 1D**, **Supplementary Table S3**). Moreover, most genes underwent CNV amplification or deletion (**Figure 1E**), with CNV significantly positively correlating with ACRG expression levels in most cancers (**Figure 1F**). Additionally, N6-methyladenosine (m6A) modification signature (42) was significantly associated with ACRG expression, suggesting a synergistic regulatory effect between m6A modification and histone acetylation modification in gene expression (**Figure 1G**). Finally, considering the prognostic significance of ACRGs, we found that ACRG expression levels were significant prognostic markers in several cancers, particularly kidney renal clear cell carcinoma and lower-grade glioma, indicating their role in malignant progression (**Figure 1H**).

### 3.2 Clustering analysis based on transcriptional expression of ACRGs across pan-cancer samples

**Figure 2:**
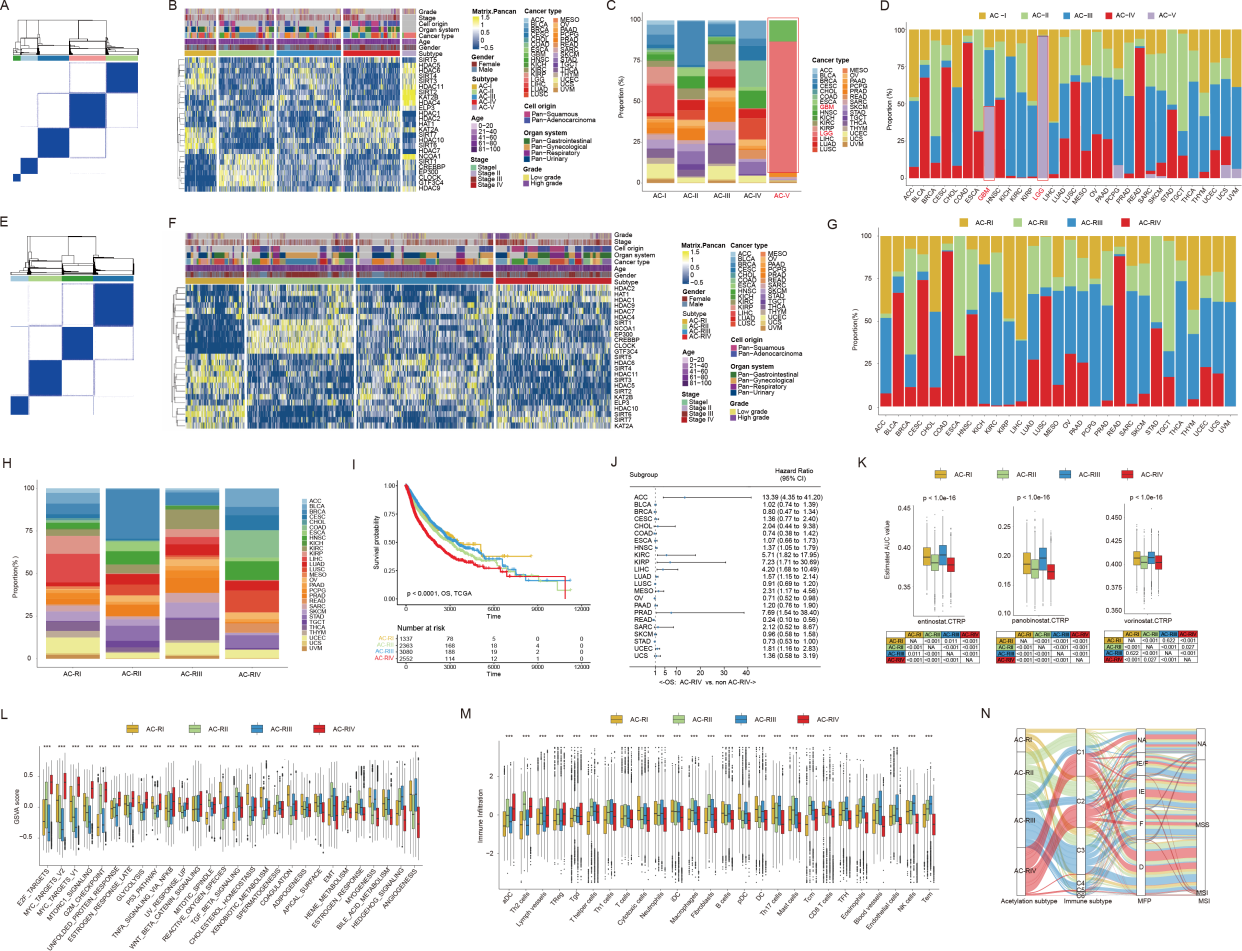
Consensus clustering based on mRNA expression of histone acetylation regulator genes (ACRGs) in the TCGA pan-cancer cohort. **(A, B)** Consensus matrix **(A)** and heatmap **(B)** depicted the expression pattern of the 25 ACRGs identified by the unsupervised clustering analysis in the TCGA pan-cancer cohort. Cohort details were illustrated as patient annotations. **(C, D)** Bar charts summarized the proportions of patients with different cancer types within and across different AC subtypes **(C)** and the proportions of patients with different AC subtypes within and across different cancer types **(D)**. **(E, F)** Consensus matrix **(E)** and heatmap **(F)** depicted the expression pattern of the 25 ACRGs identified by the unsupervised clustering analysis. Cohort details were illustrated as patient annotations. **(G, H)** Bar charts summarized the proportions of patients with different cancer types within and across different ACR subtypes **(G)** and the proportions of patients with different ACR subtypes within and across different cancer types **(H)**. **(I)** Kaplan-Meier curves of overall survival (OS) according to ACR subtypes. **(J)** The forest plot showed the associations between ACR subtypes and OS of patients in each cancer type. **(K)** Box plots exhibited predicted area under curve values of entinostat (left), panobinostat (middle), and vorinostat (right) corresponding to patients with different ACR subtypes based on CTRP analysis. **(L, M)** Box plots showed the biological pathway activation status **(L)** and tumor microenvironment landscape **(M)** among the ACR subtypes. **(N)** Sankey diagram of ACR subtypes in groups with different molecular subtypes. ***p < 0.001; NA, not available.

To explore the expression pattern of ACRGs at the pan-cancer level, we performed unsupervised clustering analysis in the TCGA pan-cancer cohort. As shown in **Figures 2A, B**, five major subtypes, denoted “AC-I” to “AC-V”, were identified. The transcriptional expression levels of 25 ACRGs among five AC subtypes are shown in **Supplementary Figure S1**. In terms of the proportion of patients with different cancer types in each AC subtype, we found that AC-V comprised over 95% of patients with glioma (up to 98% of low-grade glioma (LGG) and nearly 50% of GBM patients, **Figures 2C, D**, **Supplementary Table S4**). Moreover, AC-V subtype also exhibited the highest silhouette widths (**Supplementary Table S5**) indicating a distinctive histone acetylation status for patients with glioma, which could possibly be attributed to the unique biology of brain tissue origin, distinct oncogenic mutations, and microenvironmental pressures (43–45). Therefore, we performed further consensus clustering analysis in the remaining solid tumors of the TCGA pan-cancer cohort after eliminating gliomas. Consistent with the previous finding, four optimal clusters (**Figure 2E**, subsequently referred to as “ACR subtype”, with “R” standing for remaining tumors) were identified in this context. Each cluster displayed the enrichment of partial regulators (**Figure 2F**), contained various cancer types (**Figures 2G, H**, **Supplementary Table S6**), and exhibited similar silhouette width levels (**Supplementary Table S5**), suggesting a new classification model for solid tumors beyond the cell-of-origin context. Interestingly, among pan-gastrointestinal tumors, the proportions of esophageal squamous cell carcinoma (ESCA), stomach adenocarcinoma, colon adenocarcinoma (COAD), and rectum adenocarcinoma classified into the AC-RII subtype were 75.3%, 50.60%, 0.04%, and 0.05%, respectively, while the proportions classified into the AC-RIV subtype were 24.7%, 46.02%, 90.91%, and 87.50%, respectively, demonstrating a transition of histone acetylation modification from upper to lower gastrointestinal tract (**Supplementary Table S6**). In addition, over 50% of patients with pan-squamous cell carcinoma, except those with ESCA, were classified into AC-RIV, showing commonalities in histone acetylation among cancer types with similar tissue origin. In terms of clinical relevance, the survival analysis showed that AC-RIV subtype had the shortest overall survival (OS, **Figure 2I**) and higher mortality risks in 16 of 23 cancer types (**Figure 2J**). Furthermore, the predicted AUC values for three pan-HDACis indicated higher sensitivity in AC-RII and AC-RIV (**Figure 2K**). In terms of biological and immune characteristics (**Figures 2L, M**), we found that AC-RIV exhibited downregulated angiogenesis and estimated infiltration scores of endothelial cells but activated pathways related to cell cycle regulation (E2F, MYC, MTORC1, G2 checkpoint signaling). Finally, the Sankey diagram showed that AC-RIV patients tended to concentrate in the subtype exhibiting immune-depleted characteristics, including wound healing and IFN-gamma dominant, while AC-RIII patients were enriched in the immune-inflamed subtype (**Figure 2N**) (46, 47). Collectively, these findings suggested that the transcriptional profiling of these 25 ACRGs could aid in establishing molecular classifications transcending tissue-of-origin cancer types.

### 3.3 Clustering analysis based on the transcriptomic expression of ACRGs in patients with glioma

**Figure 3:**
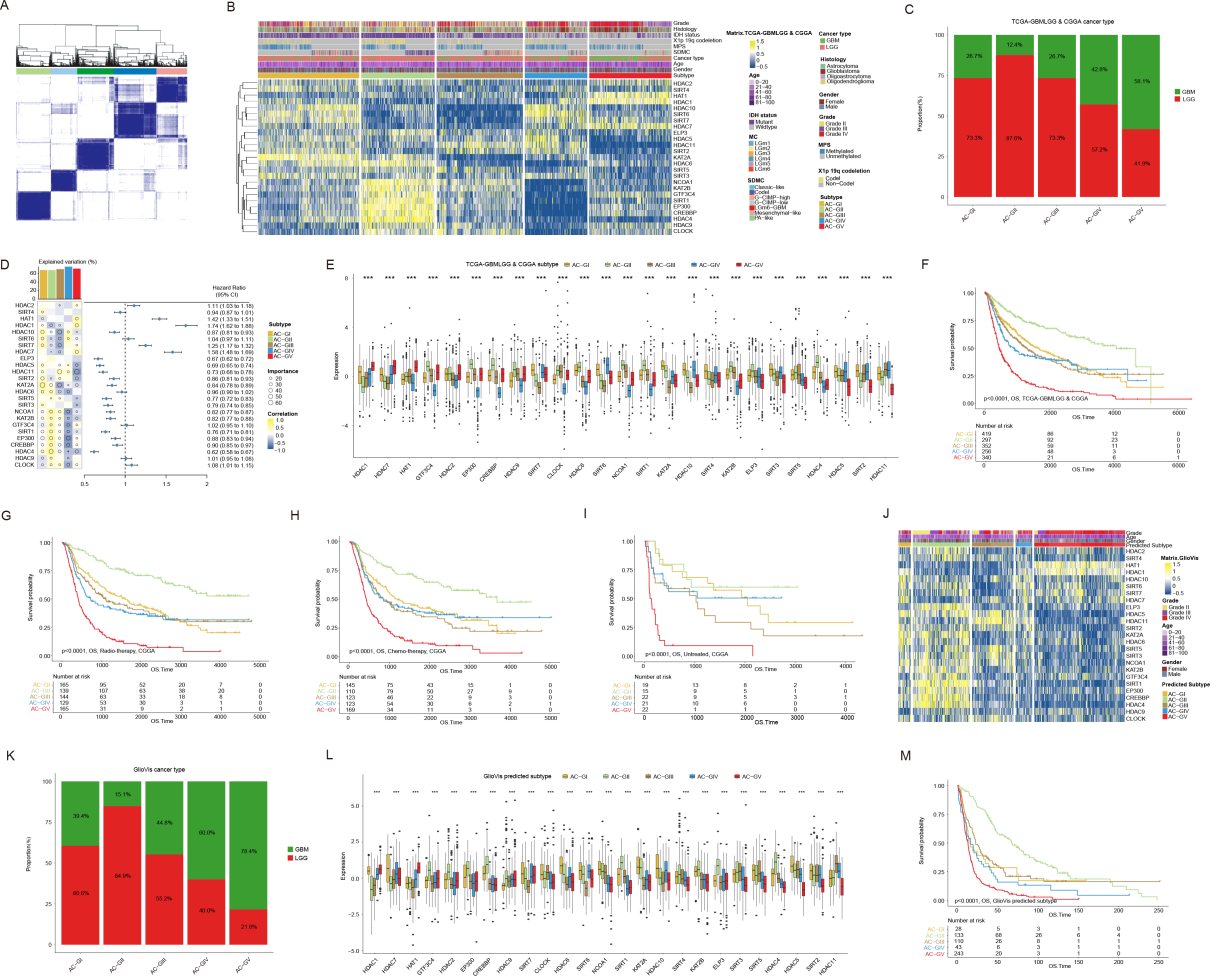
Consensus clustering based on mRNA expression of histone acetylation regulator genes (ACRGs) in patients with glioma. **(A, B)** Consensus matrix **(A)** and heatmap **(B)** depicted the expression pattern of the 25 ACRGs identified by the unsupervised clustering analysis in the TCGA-GBMLGG & CGGA cohort. Cohort details were illustrated as patient annotations. **(C)** Bar charts summarized the proportions of patients with different ACG subtypes within and across LGG and GBM. **(D)** Heatmap (left) displayed the correlation and importance generated by random forest model of each ACRGs in different ACG subtypes and the boxplot above represented the degree of interpretation of the model to the target ACG subtypes. The forest plot (right) showed the associations between different ACRGs and OS of patients. **(E)** Box plots showed the mRNA expression of 25 ACRGs in different ACG subtypes in the TCGA-GBMLGG & CGGA cohort. **(F)** Kaplan-Meier curves of overall survival (OS) according to ACG subtypes in TCGA-GBMLGG & CGGA cohort. **(G-I)** Kaplan-Meier curves of overall survival (OS) according to ACG subtypes of radio-therapied **(G)**, chemo-therapied **(H)** and untreated **(I)** patients in CGGA cohort. **(J)** Heatmap depicted the expression pattern of the 25 ACRGs between different predicted ACG subtypes in the GlioVis cohort. **(K)** Bar charts summarized the proportions of patients with different predicted ACG subtypes within and across LGG and GBM in GlioVis cohort. **(L)** Box plots showed the mRNA expression of 25 ACRGs in different predicted ACG subtypes in the GlioVis cohort. **(M)** Kaplan-Meier curves of overall survival (OS) according to predicted ACG subtypes in GlioVis cohort. ***p < 0.001.

Given the distinctive ACRG expression pattern observed in glioma patients, we integrated the transcriptome data from the TCGA-GBMLGG (containing 529 LGG and 166 GBM patients), CGGA-692 (containing 443 LGG and 249 GBM patients), and CGGA-321 (containing 182 LGG and 139 GBM patients) datasets and re-performed consensus clustering analysis. As a result, these glioma patients were classified into five clusters (subsequently referred to as “ACG subtype”, where “G” stands for gliomas), which were referred to as “AC-GI” to “AC-GV” (**Figures 3A, B**). Each cluster comprised varying proportions of LGG and GBM patients, suggesting that the ACG subtype was a novel classification scheme for glioma beyond histopathological features (**Figure 3C**). Among these clusters (**Figures 3D, E**), AC-GI was characterized by elevated expression of KAT2A, while HDAC4 emerged as the most critical ACRG for distinguishing AC-GII patients, with its high expression correlating with longer survival. For samples within AC-GIII, the synergistic downregulation of KAT2A, HDAC10, and SIRT7 mRNA expression can serve as a defining molecular characteristic. As for AC-GIV, the ACRGs that were most important for distinguishing patients within this subtype included EP300, CREBBP, NCOA1, and CLOCK, all of which exhibit low expression levels in AC-GIV. Finally, in terms of AC-GV, the mRNA expression of HDAC11 was significantly reduced, while that of HDAC1, HAT1, and HDAC7, which are strongly associated with poor prognosis, were significantly elevated in this subtype. We further explored the prognostic significance of ACG subtype in glioma patients. The results showed that, within the entire patient cohort, AC-GII subtype displayed markedly longer survival, whereas the AC-GV corresponded to worst prognosis (**Figure 3F**). Furthermore, the prognostic significance of ACG subtype was preserved in subgroup analysis stratified by dataset used (**Supplementary Figures S2A, B**) or treatment received by the patient (**Figures 3G–I**). To clarify whether our proposed ACG classification scheme is robust, we collected supplementary transcriptomic data of glioma samples from the GlioVis database and GEO database (GSE43378). Subsequently, we classified the patients using the NTP algorithm based on the molecular expression signatures obtained by PAM algorithm in the whole transcriptome (48) (**Supplementary Figures S2C-E**). Similarly, we identified five patient groups based on ACRG transcription expression in both GlioVis and GSE databases, and the ACRG expression characteristics (**Figures 3J, L**, **Supplementary Figures S2F, G**), histopathological features (**Figure 3K**) and prognostic differences (**Figure 3M**, **Supplementary Figure S2H**) between each group were generally consistent with the original ACG subtype. Taken together, these results indicated that the transcriptional expression of ACRGs represents a novel molecular marker with significant clinical implications for the precise characterization and classification of gliomas.

### 3.4 Association between ACG subtypes and other genetic markers with clinical significance

**Figure 4:**
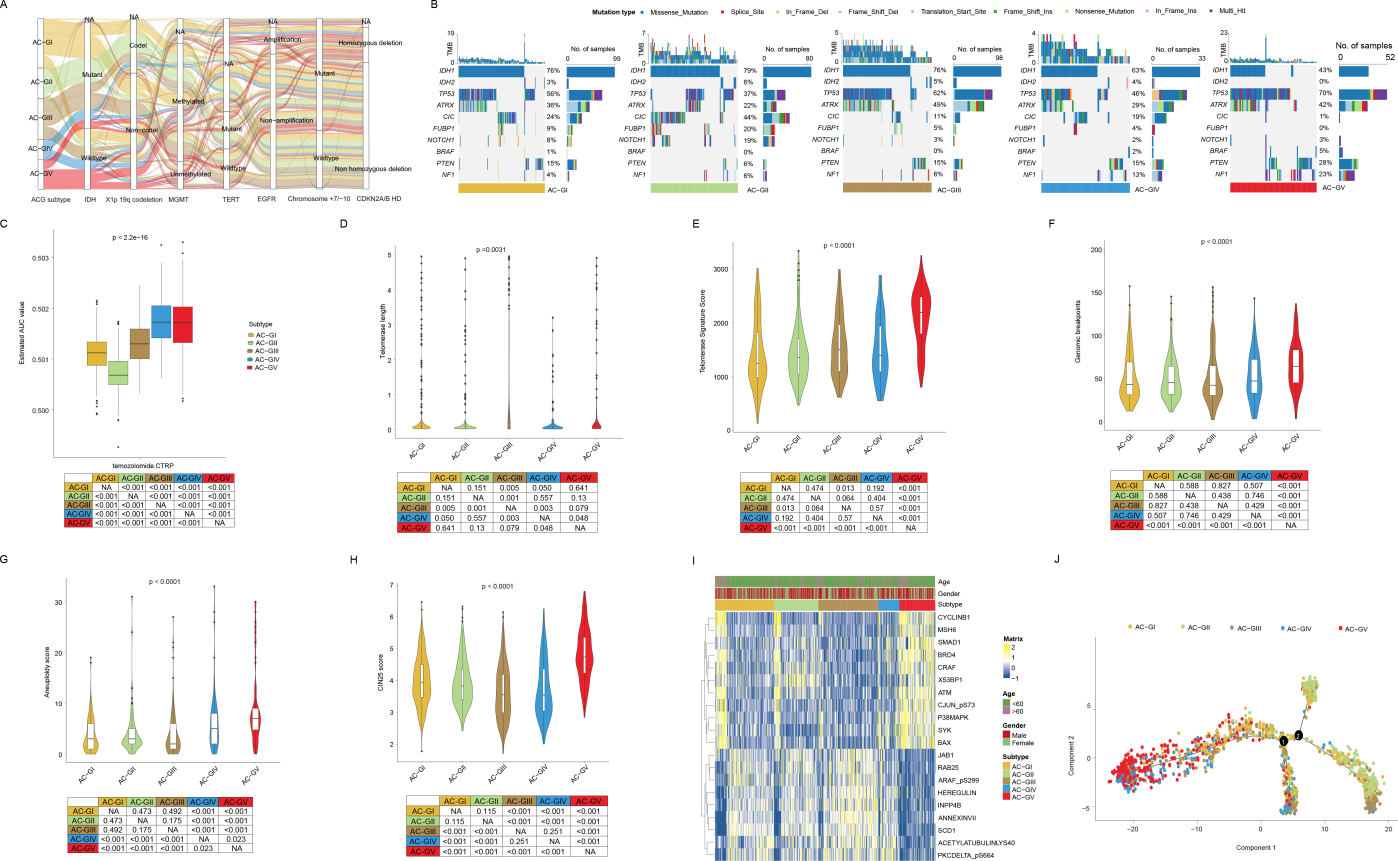
The association between ACG subtypes and other genetic markers with clinical significance. **(A)** Sankey diagram of ACG subtypes in groups with different molecular subtypes in the TCGA-GBMLGG cohort. **(B)** Oncoprints depicted significantly mutated genes and different mutation types of different ACG subtypes in the TCGA-GBMLGG cohort. **(C)** Box plot showed the predicted area under curve values of temozolomide based on the CTRP database. **(D-H)** Violin plots displayed the telomere length **(D)**, telomerase signature score **(E)**, genomic breakpoints **(F)**, aneuploidy score **(G)**, and CIN25 score **(H)** among different ACG subtypes in TCGA-GBMLGG cohort. **(I)** Heatmap exhibited the landscape of differentially expressed proteins in different ACG subtypes in the TCGA-GBMLGG cohort. **(J)** Pseudotemporal analysis plot of TCGA-GBMLGG & CGGA cohort demonstrated the potential development trajectory of different ACG subtypes. NA, not available.

Currently, the clinical classification of glioma has been achieved by integrating both histopathologic characteristics and molecular markers, such as IDH mutation status, codeletion of chromosomal arms 1p and 19q (1p/19q codeletion), and combined gain of chromosome 7 and loss of chromosome 10 (7+/10-), etc (49). Hence, we investigated the correlation between ACG classification and molecular markers that are routinely assessed in clinical practice. As shown in **Figures 4A, B**, we observed that the AC-GV subtype was significantly enriched with molecular features linked to high malignancy, such as IDH wild-type, 1p/19q non-codeletion, EGFR amplification, 7+/10- mutations, and CDKN2A/B homozygous deletion (HD) (**Supplementary Table S7**). In addition, some genetic mutations that were frequently observed in GBM, including PTEN and NF1, were more prevalent in AC-GV compared to other ACG subtypes. Conversely, the AC-GII subtype was concentrated in molecular characteristics such as IDH mutant, 1p/19q codeletion, non-EGFR amplification, and CDKN2A/B non-HD. Notably, the CIC mutation, a genetic feature of oligodendrogliomas, exhibited the highest incidence in the AC-GII subtype. Furthermore, the frequency of MGMT promoter methylation, a biomarker indicative of response to temozolomide, was the highest in the AC-GII (85.4%), followed by AC-GIII, whereas AC-GV showed the lowest incidence of MGMT methylation. Consistently, the drug sensitivity analysis also showed that the predicted AUC for temozolomide was the lowest in AC-GII but significantly higher in AC-GIV and AC-GV compared to other subtypes, suggesting that patients within AC-GIV and AC-GV may have primary resistance to temozolomide (**Figure 4C**). Alterations in telomeres and telomerase have been regarded as prognostic biomarkers for glioma patients (50). Our findings revealed that the telomerase signature score and telomere length was the highest in AC-GV subtype (**Figures 4D, E**). However, aberrant telomere variant repeats and targeted telomere insertion did not exhibit significant differences among the various ACG subtypes (**Supplementary Figure S3**). Additionally, we also evaluated the associations between ACG subtypes and genomic instability. The findings indicated that genomic breakpoints, aneuploidy scores, and chromosomal instability 25 scores were significantly elevated in AC-GV patients compared to other subtypes (**Figures 4F–H**). Correspondingly, the proteomic data analysis also revealed increased expression of proteins involved in DNA damage repair, such as ATM, X53BP1, and MSH6, within the AC-GV subtype (**Figure 4I**). Finally, the transcriptional differentiation trajectory analysis showed that the AC-GV subtype was concentrated at the terminal point of the differentiation trajectory, indicating that AC-GV represents the most aggressive subgroup of glioma (**Figure 4J**).

### 3.5 Disparity in biological characteristics and immune TME among ACG subtypes

**Figure 5:**
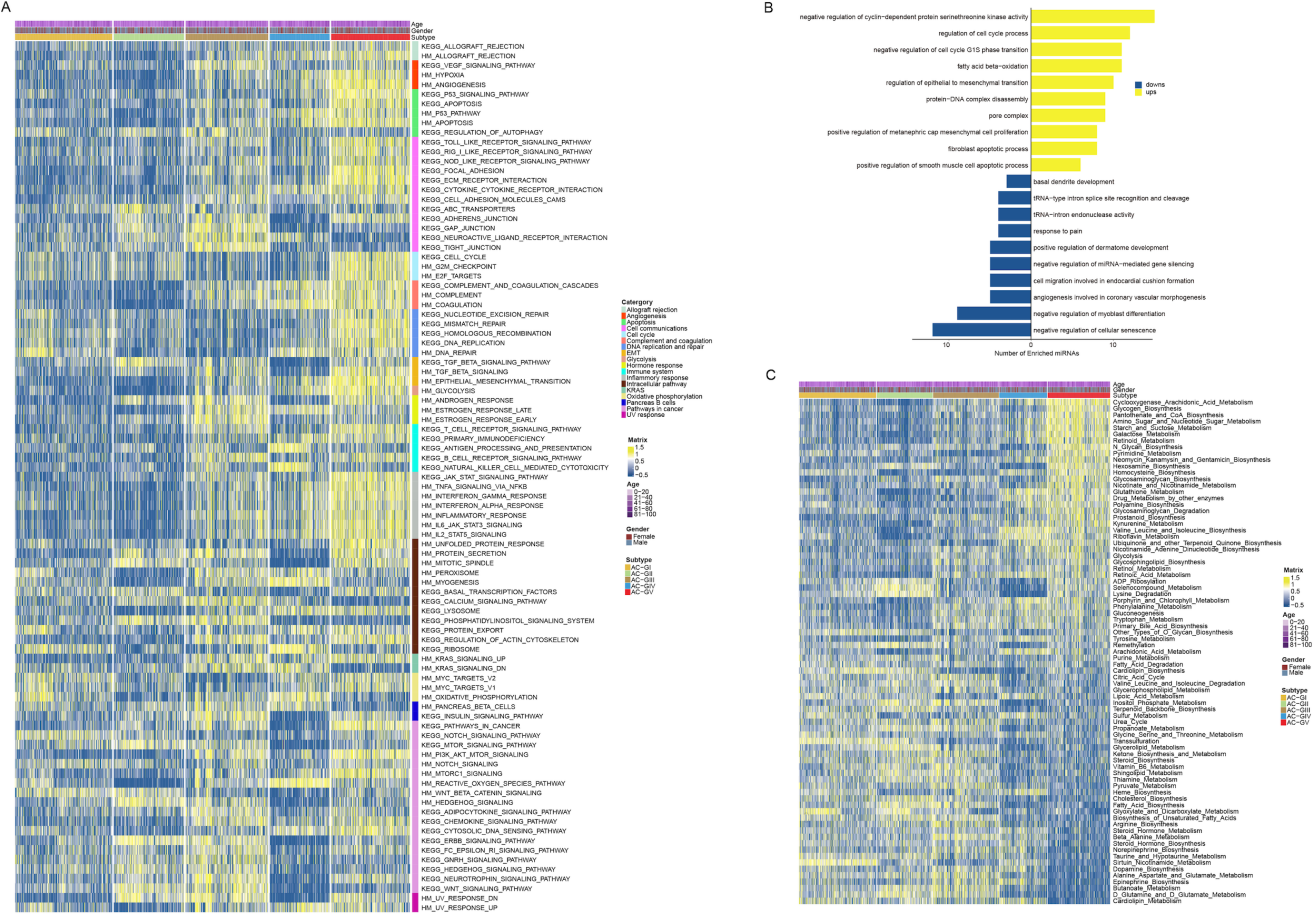
Disparity in biological and metabolic characteristics between ACG subtypes. **(A)** Heatmap depicted the Hallmark and KEGG biological pathways among 5 ACG subtypes. **(B)** Bar plots showed the results of the over-representation analysis performed by using the up-regulated and the down-regulated microRNAs derived by the differential expressed miRNA analysis. **(C)** Heatmap displayed the metabolic characteristics among 5 ACG subtypes.

The biological characteristic of each ACG subtype was assessed using GSVA analysis (**Figure 5A**, **Supplementary Figures S4A, B**). The results suggested that the majority of biological pathways cataloged in Hallmark and KEGG databases showed high activation levels in AC-GV subtype. Specifically, biological processes related to angiogenesis, epithelial-mesenchymal transition, unfolded protein response, MTOR signaling, cell cycle regulation, DNA damage repair, and apoptosis had the highest activation levels in AC-GV compared to other subtypes. Similarly, pathway enrichment analysis based on differential miRNAs also indicated that miRNAs related to epithelial-mesenchymal transition and cell cycle regulation were significantly upregulated in patients with AC-GV compared with others (**Figure 5B**). The most significantly elevated pathways in the AC-GIV subtype were the ROS pathway, oxidative phosphorylation, and myogenesis, indicating a higher degree of oxidative stress in the tumor tissues of AC-GIV. Interestingly, the activation levels of the above three biological processes were found to be significantly inhibited in AC-GII subtype. The enrichment of metabolism-related pathways in each ACG subtype is shown in **Figure 5C**, **Supplementary Figure S4C**. Notably, AC-GV was characterized by the highest activation levels of glycolysis, indicating that targeting glucose metabolism could also be a potential therapeutic strategy for these patients.

**Figure 6:**
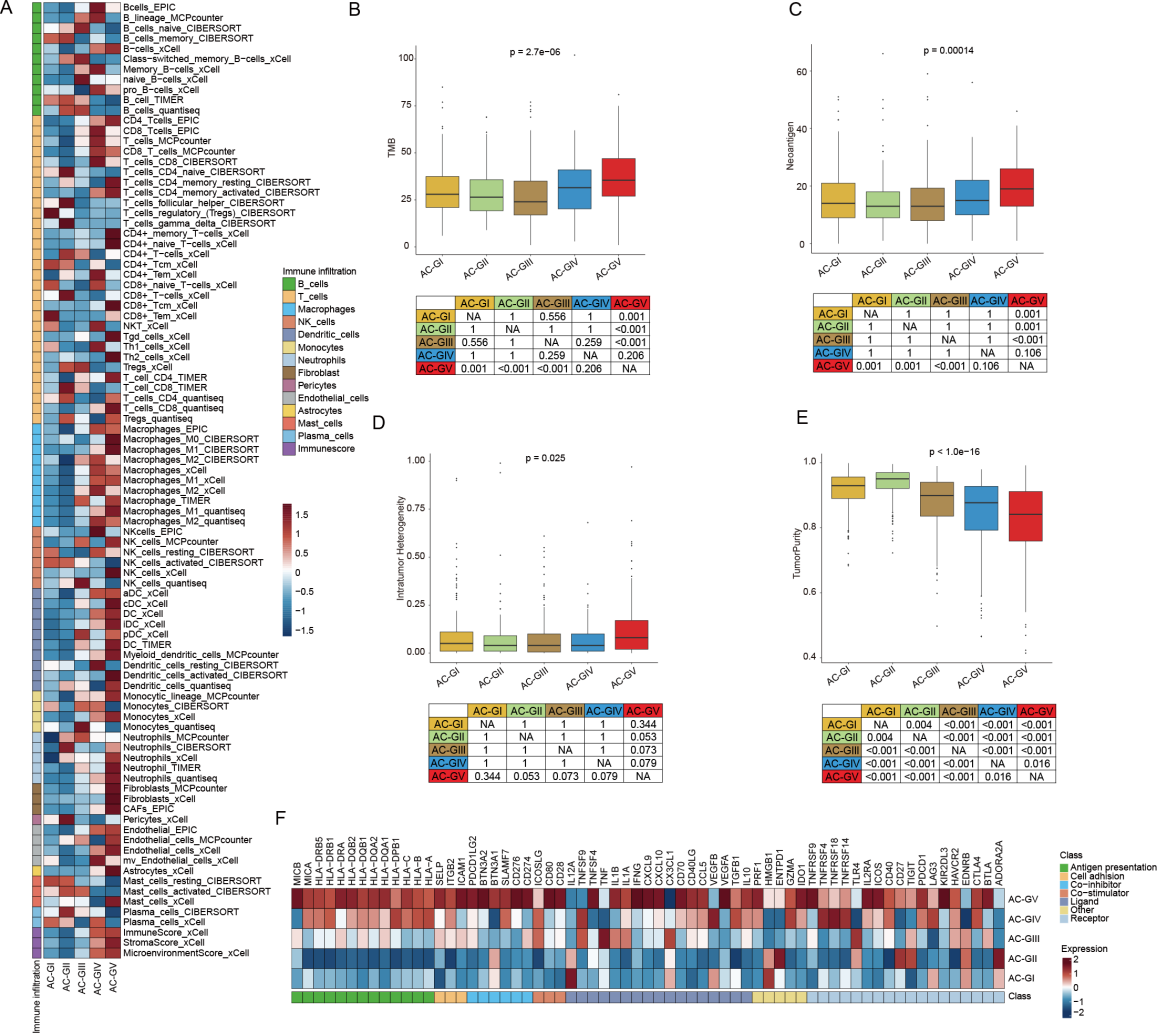
Diversity in immune tumor microenvironment between ACG subtypes. **(A)** Heatmap displayed the tumor microenvironment cell infiltration among 5 ACG subtypes. **(B-E)** Box plots illustrated the differences in TMB score **(B)**, Neoantigen score **(C)**, Intratumor Heterogeneity score **(D)**, and TumorPurity score **(E)** between 5 ACG subtypes. **(F)** Heatmap showed expression of different immune regulatory pathways genes in different ACG subtypes. NA, not available.

To explore the immune heterogeneity among different ACG subtypes, we estimated the immune infiltration score of each sample utilizing six transcriptomic-based immune cell infiltration algorithms embedded in the IOBR package (21) (**Figure 6A**, **Supplementary Figure S5A**). The analysis revealed that AC-GV patients, despite having the poorest prognosis, exhibited the highest estimated enrichment score of TME cells, especially CD4^+^ T cells, M1 macrophages, activated dendritic cells, and cancer-associated fibroblasts (CAFs). In particular, CAFs exhibit a highly infiltrated pattern that was specific in AC-GV. Consistent with findings generated from immune cell infiltration analysis, patients within AC-GV also exhibited the highest levels of TMB (**Figure 6B**), neoantigens (**Figure 6C**), and intratumoral heterogeneity (**Figure 6D**), along with the lowest levels of tumor purity (**Figure 6E**). Additionally, the activation levels of immune regulatory pathway genes (e.g., antigen presentation, chemokine, and immune checkpoint, etc) were also the highest in AC-GV (**Figure 6F**, **Supplementary Figure S5B**). On the contrary, for patients within AC-GI and AC-GII subtypes, both the estimated infiltration abundance of most immune cells and the expression levels of immune-related genes were observed to be at low levels, suggesting that the TME of these two subtypes presents an “immune desert” phenotype. In brief, the results mentioned above identified an extensive immune infiltration disparity among five ACG subtypes within gliomas.

### 3.6 Screening of potential therapeutic agent by CTRP and CMap database analysis

**Figure 7:**
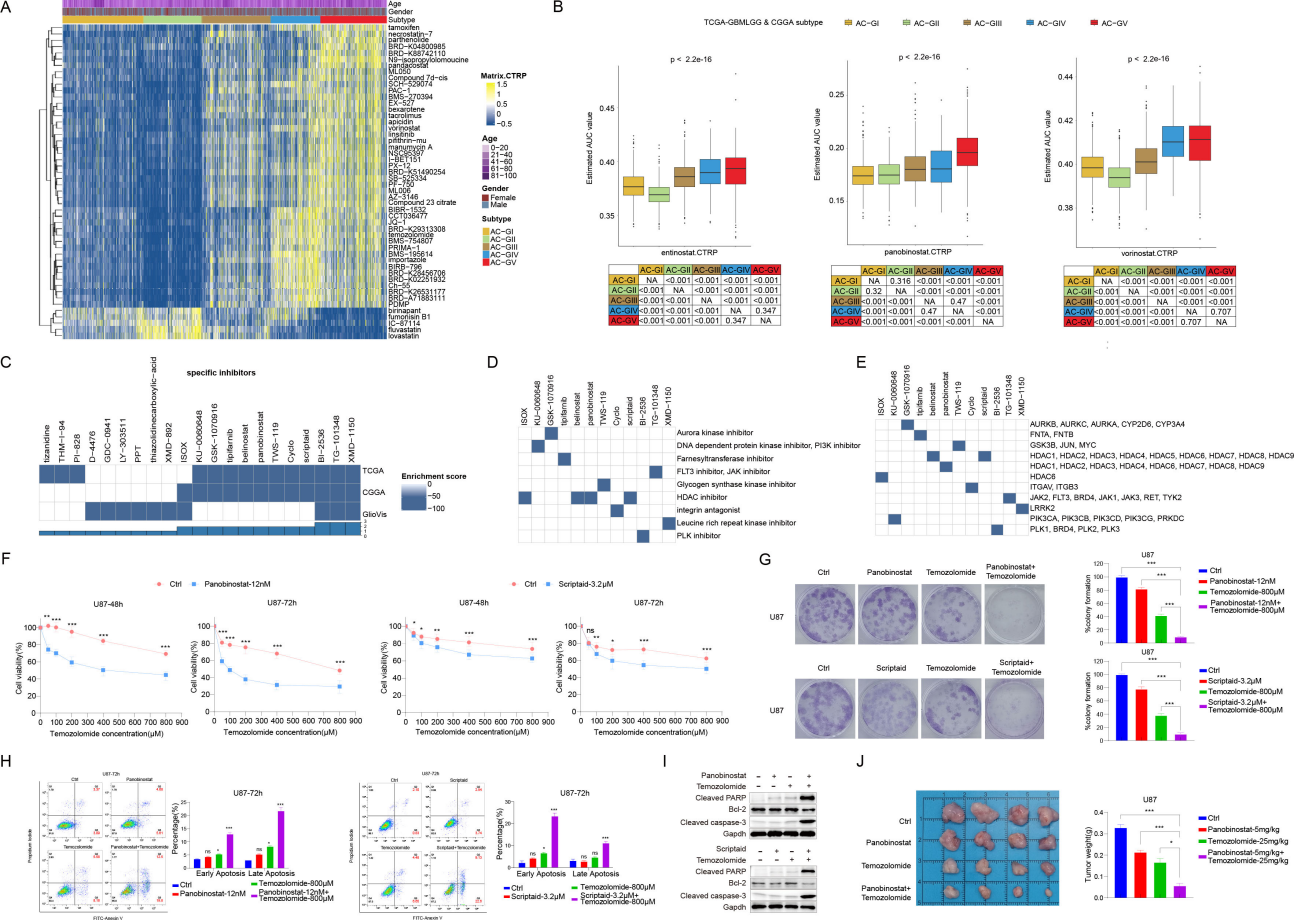
Screening of potential therapeutic agent by CTRP and CMAP database analysis. **(A)** Heatmap depicted the predicted area under curve values of 30 drugs with the most significant difference among different ACG subtypes based on the CTRP database. **(B)** Box plot showed the predicted area under curve values of entinostat (left), panobinostat (middle), and vorinostat (right) in different ACG subtypes based on the CTRP database. **(C)** Heatmaps showing the enrichment score of each compound based on the Connectivity Map analysis in the TCGA-GBMLGG, CGGA, and GlioVis cohorts. **(D, E)** Heatmap showing the mechanisms of the action **(D)** and gene targets of each compound **(E)**. **(F)** U87 cells were treated with vehicle control + Temozolomide, Panobinostat (12 nM) + Temozolomide, and Scriptaid (3.2 μM) + Temozolomide for 48h or 72h. Cell viability was detected by cell counting kit-8 (CCK8). **(G)** U87 cells were treated with vehicle control, Panobinostat(12nM), Scriptaid(3.2μM), Temozolomide(800μM), Panobinostat(12nM) and Temozolomide(800μM), or Scriptaid(3.2μM) and Temozolomide(800μM) for 48h, and cultured up to 2 weeks in drug-free DMEM complete media. Colonies were fixed with paraformaldehyde and stained with crystal violet, then analyzed by ImageJ. Representative images of colony formation assay and mean percent difference in colony formation in different drug treatment groups from three independent experiments. **(H)** Representative Annexin V and PI staining of vehicle control, Panobinostat, Scriptaid, Temozolomide, Panobinostat + Temozolomide, and Scriptaid + Temozolomide treated U87 cells. Quantification (percentage of) early and late apoptotic cells. **(I)** U87 cells were incubated with the drugs mentioned above for 48h, and lysates were subjected to Western blot to identify the levels of apoptotic proteins, cleaved PARP, Bcl2 and cleaved caspase-3. **(J)** U87 cells (1X10^6 cells in 100μl of DMEM) were intracranially injected into 6 week-old female BALC/c nu/nu mice. After one week, measured tumor growth, and mice were randomized into 4 groups and treated with vehicle control, Panobinostat(5mg/kg), Temozolomide(25mg/kg), Panobinostat(5mg/kg) +Temozolomide(25mg/kg) for 5 days a week. On the 14th day, images of tumors in both groups of mice were obtained. Histogram comparison of tumor weights among different treatment groups in mice with tumors. *p < 0.05, **p < 0.01, ***p < 0.001. ns, not significant.

To explore the potential therapeutic agents that could be used for treating glioma patients within different ACG subtypes, we calculated the AUC value of 354 drugs documented in CTRP database among TCGA-GBM and CGGA patients (**Figures 7A, B**). We discovered that in addition to temozolomide mentioned earlier, some pan-HDACis (vorinostat and pandacostat) also showed the lowest mean AUC values in AC-GII subtype, while the average AUC values were significantly increased in AC-GV, suggesting that patients within AC-GII rather than AC-GV subtype may benefit from pan-HDAC inhibitor treatment. Interestingly, certain statins (e.g., fluvastatin and lovastatin), which have demonstrated anti-tumor efficacy in both *in vivo* and *in vitro* studies (51), showed significantly lower mean AUC values in AC-GV patients compared to patients with other ACG subtypes, suggesting that AC-GV patients may be suitable for statin therapy.

Since patients in the AC-GII subtype tended to be more sensitive to temozolomide, we used the CMap bioinformatic tool to identify drug candidates that can improve the efficacy of temozolomide based on the differential transcriptome characteristics between AC-GV and AC-GII subtypes. As shown in **Figure 7C**, we identified 12 compounds whose CMap scores were lower than -90 in at least two glioma cohorts. Further investigation of the activity and gene targets of these compounds revealed that four compounds were inhibitors of the HDAC family (**Figures 7D, E**). Among them, belinostat, panobinostat, and scriptaid were pan-HDACis, while ISOX is capable of specifically inhibiting the activity of HDAC6. Since our previous findings have demonstrated the lowest sensitivity of pan-HDACis and temozolomide in AC-GV type patients (**Figure 7B**), we hypothesized whether the combination of pan-HDACis and temozolomide could exhibit synergistic antitumor effect. As panobinostat and scriptaid exhibited lower enrichment scores compared to the other identified pan-HDACis, we selected panobinostat and scriptaid for subsequent empirical validation through wet lab experiments. By conducting *in vitro* experiment, we validated that panobinostat or scriptaid could enhance the inhibitory effects of temozolomide on U87 cell proliferation (**Figure 7F**) and colony formation (**Figure 7G**), as well as potentiate the temozolomide induced cell apoptosis (**Figures 7H, I**). The xenotransplantation experiments further indicated that temozolomide combined with panobinostat therapy limited the capacity of U87 cells to form tumors *in vivo* (**Figure 7J**). To conclude, these findings suggested that panobinostat or scriptaid could serve as chemosensitizers for AC-GV subtype. The combination of pan-HDACis with temozolomide represents a promising novel therapeutic strategy for patients with AC-GV subtype.

### 3.7 Construction and validation of an ACG subtype-related scoring model to discern AC-GII and AC-GV

**Figure 8:**
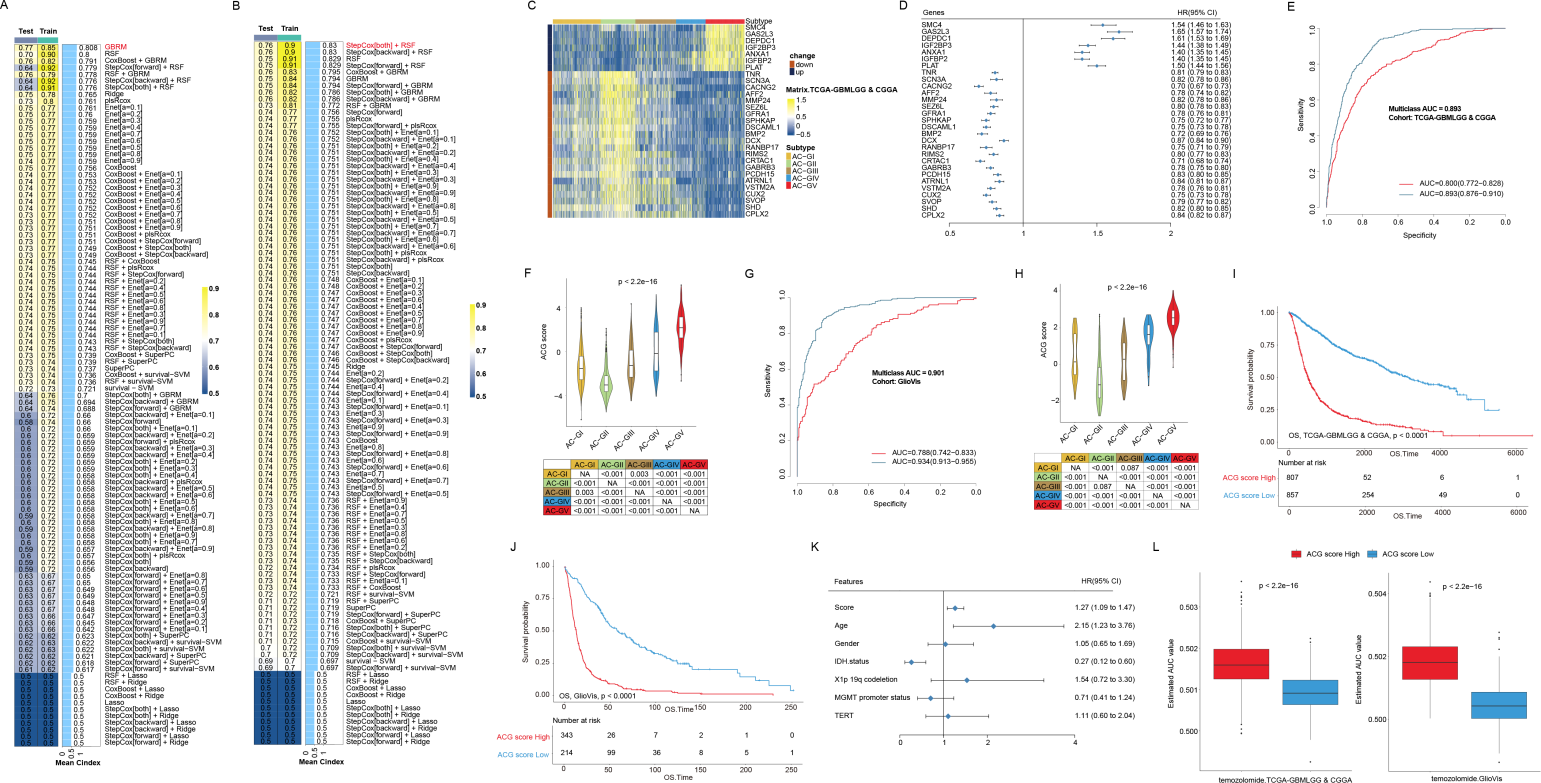
Construction and validation of ACG score in different glioma cohorts. **(A, B)** Bar plots displayed machine learning prediction models using differentially expressed genes in AC-GII **(A)** and AC-GV subtype **(B)** respectively via leave-one-out cross validation framework and further calculated the C-index of each model across Train and Test datasets in TCGA-GBMLGG & CGGA cohort. **(C)** Heatmap exhibited the expression of genes consisting of ACG score in different ACG subtypes in the TCGA-GBMLGG & CGGA cohort. **(D)** The forest plot showed the associations between the expression of genes consisting of ACG score and OS of patients in the TCGA-GBMLGG & CGGA cohort. **(E, F)** The receiver operating characteristics curve **(E)** and violin plots **(F)** showed the ability of ACG score to distinguish AC-GV (blue) and AC-GII subtype (red) from other ACG subtypes in the TCGA-GBMLGG & CGGA cohort. **(G, H)** The receiver operating characteristics curve **(G)** and violin plots **(H)** showed the ability of ACG score to distinguish predicted AC-GV (blue) and predicted AC-GII subtype (red) from other ACG subtypes in the TCGA-GBMLGG & CGGA cohort. **(I, J)** Kaplan-Meier curves of OS in the TCGA-GBMLGG & CGGA cohort **(I)** and GlioVis cohorts **(J)** according to the ACG score. **(K)** The forest plot showed the associations between the various factors and OS in a multivariate regression model in the TCGA-GBMLGG cohort. **(L)** Box plots exhibited predicted area under curve values of temozolomide among different ACG score groups based on CTRP analysis in the TCGA-GBMLGG & CGGA (left) and GlioVis (right) cohorts. NA, not available.

Since AC-GII and AC-GV were characterized by possessing contrasting clinical characteristics, with AC-GII patients having improved prognosis and higher temozolomide sensitivity, while AC-GV displayed the opposite features, it would be beneficial to establish an effective scoring scheme capable of accurately identifying AC-GII and AC-GV patients. To provide a more comprehensive view of the acetylation landscape without overlooking important genes that are not directly involved in enzymatic process, we screened the candidate genes from the whole transcriptome. To reduce the dimensions of DEGs in AC-GII (**Figure 8A**, **Supplementary Table S8**) and AC-GV (**Figure 8B**, **Supplementary Table S9**), we employed LOOCV framework to fit 105 prediction models and computed the c-index for each within the TCGA/CGGA cohort. The GBRM algorithm was selected for AC-GII subtype discrimination, while the combination of StepCox and RSF was chosen to identify the AC-GV subtype. Subsequently, by combing the results of these two prediction models, we constructed a scoring scheme composed of 29 genes (**Supplementary Table S10**), termed the “ACG score”, which was defined as (the average value of up-regulated genes in AC-GV) - (the average value of up-regulated genes in AC-GII). Among these genes, SMC4, CAS2L3, DEPDC1, IGF3BP3, ANXA1, IGFBP2, and PLAT were significantly upregulated in the AC-GV subtype and associated with higher survival risks, while the remaining 22 genes were upregulated in the AC-GII subtype and linked to improved prognosis (**Figures 8C, D**). To examine the distribution of 29 scoring genes among different TME cells, we obtained the glioma scRNA-seq dataset GSE182109, in which 214,366 cells from 44 tumor tissues were categorized into seven major cell types, including glioma cells, oligodendrocytes, myeloid cells, T cells, B cells, endothelial cells, and pericytes (**Supplementary Figures S6A, B**). The marker gene expression for different cell types was shown in **Supplementary Figure S6C**. The violin plot demonstrated that IGFBP2, SMC4, PLAT, and ANXA1, showed relatively higher expression levels in pericytes and glioma cells compared to other scoring genes (**Supplementary Figure S6D**). The ROC curves demonstrated that the ACG score could accurately identify both the AC-GII and AC-GV subtypes, with elevated ACG scores indicating AC-GV and reduced scores prompting AC-GII subtype in TCGA-GBMLGG&CGGA (**Figures 8E, F**), GlioVis cohorts (**Figures 8G, H**), and GSE43378 (**Supplementary Figures S7A, B**). Subsequent survival analyses revealed that elevated ACG score was linked to higher mortality risk in the univariate model (**Figures 8I, J**, **Supplementary Figure S7C**) and was validated as an independent prognostic indicator by multivariate Cox regression (**Figure 8K**, **Supplementary Tables S11**-**13**). Moreover, high ACG score subgroup was associated with reduced temozolomide sensitivity (**Figure 8L**, **Supplementary Figure S7D**). We also investigated the correlation between ACG score and molecular markers that are routinely assessed in clinical practice. As shown in **Supplementary Figure S8**, we observed that the high ACG score group was enriched with molecular features linked to high malignancy, such as IDH wild-type, 1p/19q non-codeletion, EGFR amplification, 7+/10- mutations, and CDKN2A/B HD (**Supplementary Table S14**). Meanwhile, the frequency of MGMT promoter methylation, a biomarker indicative of response to temozolomide, was significantly lower in the high ACG score group (82.1%). Taken together, these findings confirmed the robustness and reproducibility of the ACG subtypes and revealed that the ACG score could be a reliable biomarker predicting survival outcomes and therapeutic efficacy in glioma patients.

## 4 Discussion

Growing evidence has demonstrated that histone acetylation plays a pivotal role in tumorigenesis and progression owing to its indispensable function in dynamic transcriptional control and the maintenance of genomic integrity (52). Therefore, enzymes involved in the histone acetylation process, particularly members of the HDAC family, are considered a promising class for drug targets (53). Currently, most developed HDACis simultaneously target multiple classes of enzymes, including classes I, II, and IV (54). Although these pan-HDACis were found to exert potent anti-tumor effects both *in vivo* and *in vitro* (55, 56), their clinical efficacy was observed predominantly in patients with hematological tumors (53), failing to meet expectations in the treatment of most solid tumors (10, 57, 58). In this study, by performing a clustering analysis based on transcriptional expression of ACRG in the pan-cancer cohort, we found that compared to other tumor types, which tend to cluster together due to similarity in ACRG transcriptional expression patterns, the ACRG expression characteristics of glioma patients have strong cancer species specificity; more than 90% of patients in the AC-V subtype are diagnosed with glioma, while each of the other four subtypes contains over 15 tumor types. Moreover, each AC subtype was characterized by the distinct expression pattern of histone acetylation-modifying enzymes. This finding suggested that the mRNA expression characteristics of ACRG were able to classify tumors beyond their anatomical origin. Meanwhile, such heterogeneous distribution pattern implies that clinical trials of HDACis based solely on the conventional anatomical classification of cancer, as currently performed in pan-HDACi clinical trials, may fail to effectively stratify patients who may experience benefits, possibly resulting in suboptimal outcomes. Particularly, we noticed that patients with COAD were predominantly distributed to the AC-IV and AC-RIV subtypes (over 90%), indicating a homogeneous acetylation modification profile within this population. Recently, Wang et al. reported the significant therapeutic benefits of combining a PD-1 antibody, an HDACi, and a VEGF antibody in treating patients with microsatellite stable/proficient mismatch repair advanced colorectal cancer (59). Given that most patients with COAD were categorized into the AC-RIV subtype, the proposed triplet combination therapy may also yield positive outcomes in non-colorectal AC-RIV patients.

Another important finding of this study is that we proposed a classification model for gliomas based on ACRG transcriptional expression patterns. In recent years, the importance of molecular alterations in achieving precise classification of gliomas has been widely recognized. Some molecular markers, such as IDH, 1p/19q codeletion, EGFR amplification, CDKN2A/B loss, and TERT promoter mutation, etc. have been routinely tested in clinical practice and cooperated with histopathological information to form the classification scheme of glioma (49). However, the intra-subtype heterogeneity still exists under the current categorizations of gliomas, suggesting that more molecular markers need to be developed for more detailed classification. To our knowledge, this is the first report exploring the role of transcriptional expression of ACRGs in classifying gliomas. Our study showed that differences exist in the distribution of transcriptional expression of ACRG in glioma, and this difference is independent of some classical glioma classification indicators, such as IDH, etc. Among them, AC-GII patients, who were characterized by high expression of HDAC4, EP300, and CREBBP, had the best prognosis and were sensitive to temozolomide and pan-HDACis. HDAC4 is a member of the Class IIa HDAC family that is highly expressed in brain cells. Although analysis of multiple bulk RNA-seq datasets of glioma patients showed that higher mRNA expression of HDAC4 was significantly correlated with lower tumor grade and prolonged OS (60), overexpression of HDAC4 (especially its nuclear overexpression) promotes proliferation, invasion, and drug resistance in glioma cells in cellular experiments (61, 62). Therefore, we hypothesized that the biological role of HDAC4 *in vivo* may be modulated by additional genetic factors (e.g., gene mutations) rather than being independent. Unlike HDAC4, EP300 and CREBBP are acyltransferases whose activity is essential for promoting histone H2B hyperacetylation. The role of histone H2B hyperacetylation in tumorigenesis and development remains controversial (63, 64). In gliomas, the degree of H2B acetylation has been reported to be associated with reduced immune infiltration scores. Coincidentally, our results also indicated that the estimated abundance of immune cell infiltration within the TME of AC-GII patients is notably low. This observation implies that the use of inhibitors targeting EP300 and CREBBP molecules may help to heat the TME of AC-GII tumors and exert a synergistic effect with anti-PD-1 therapy in patients belonging to this subtype.

In contrast to AC-GII, AC-GV patients, characterized by high expression of HAT1 and HDAC1 combined with loss of HDAC11 expression, had the worst prognosis and the highest predicted AUC values for temozolomide and pan-HDACis, suggesting that these patients may have primary resistance to these drugs. Meanwhile, AC-GV subtype displayed enriched genomic instability features, including genomic breakpoints, aneuploidy, and intratumor heterogeneity. In terms of estimated immune infiltration scores, although the AC-GV tumors had a high enrichment of CD8T cells maker gene expression, CAFs, which have been recognized as crucial contributors to the physical obstruction of T-cell infiltration through the formation of a dense extracellular matrix, also exhibited highly estimated infiltration abundance specifically in this subtype (65). Therefore, we hypothesized that the TME characteristics of AC-GV could be cataloged into the “immune-excluded” phenotype, which may account for the poor prognosis of AC-GV subtype. Interestingly, through the CTRP database analysis, we found that these patients were more sensitive to statins. Previous studies have reported that statins suppressed glioma growth through various mechanisms *in vitro* (66); however, clinical observational studies showed that the use of statins only reduces the incidence rate of glioma but does not prolong the survival of glioma patients (67). Our study suggested that only patients within AC-GV subtype may benefit from statin therapy. Therefore, it is necessary to screen enrolled patients for clinical studies on statins. In addition, through CMap analysis based on DEGs between AC-GV and AC-GII, we also uncovered four HDACis as potential chemotherapy sensitizers. Among them, we further validated that the pan-HDAC inhibition by either panobinostat or scriptaid demonstrated the ability to enhance chemosensitivity to temozolomide in GBM cells. The combination therapy was more effective in suppressing cell proliferation and inducing apoptosis than either treatment alone. Since the dysregulation of HDACs has been implicated in tumor adaptation and resistance to genotoxic chemotherapy (68, 69), the pairment of HDACis with DNA-damaging chemotherapeutic agents in GBM treatment appears to be a rational and promising treatment sensitizing option for AC-GV patients, thus warrant further investigation of this combination therapy in clinical trials. Moreover, the ACG scoring model was composed of 29 genes, which is easy to be converted into a clinically usable kit, making the generation and standardization of ACG score simple and convenient. The ACG score was not only an independent predictor of poor prognosis of glioma patients, but could also effectively identify patients with temozolomide resistance, guide the decision of the combination therapy of pan-HDACis and temozolomide, and thus possess significant clinical transformation values.

This study has some limitations. First, previous studies have reported that the ACRGs included in this study regulate many other acylation modifications besides acetylation, including propionylation, crotonylation, butyrylation, succinylation, glutarylation, 2-hydroxyisobutyrylation, and β-hydroxybutyrylation (70–75). Therefore, the connection between tumor classification system we established based on the transcriptional expression of ACRGs and the levels of histone acetylation modifications should be interpreted with caution. Second, our study was solely based on a retrospective analysis of public patient cohorts. Therefore, it is necessary to collect multi-center patient cohorts to verify our findings. Further validation in prospective cohorts using patient specimens can significantly enhance the clinical applicability of ACG score. Third, the cut-off values of the ACG score need to be standardized in future prospective studies. Fourth, the analysis of drug sensitivities was based exclusively on in silico results, thus, requiring further wet lab experiment validation in the future. Last, other identified potential sensitizers for temozolomide combination treatment require further experimental verification.

In conclusion, our study suggested that the transcriptional expression characteristics of HATs and HDACs can yield novel molecular classifications that transcend tissue-of-origin cancer types. In particular, we identified five novel molecular subtypes (ACG subtype) in the context of gliomas, unveiling differences in clinical, biological, TME, and genomic alteration characteristics. These classification patterns and the associated ACG score established in this study may serve as key references for individualized treatment of gliomas.

## Data Availability

The data supporting the findings of this study are deposited in “TCGA pan-cancer” section of University of California Santa Cruz (UCSC) Xena database (https://xenabrowser.net), GEO (http://www.ncbi.nlm.nih.gov/geo), CGGA database (http://www.cgga.org.cn/), and GlioVis (http://gliovis.bioinfo.cnio.es/) database.

## Glossary

ACRGs: histone acetylation regulator genes
AUC: area under the dose-response curve
CGGA: Chinese Glioma Genome Atlas
CMap: Connectivity Map
CNV: copy number variation
CTRP: Cancer Therapeutics Response Portal
COAD: colon adenocarcinoma
DEGs: differentially expressed genes
ESCA: esophageal squamous cell carcinoma
GBM: glioblastoma
GBRM: Generalized Boosted Regression Modeling
GSVA: gene set variation analysis
HATs: histone acetyltransferases
HD: homozygous deletion
HDACs: histone deacetylases
HDACis: histone deacetylase inhibitors
IDH: isocitrate dehydrogenase
LGG: lower-grade glioma
LOOCV: leave-one-out cross-validation
M6A: N6-methyladenosine
NTP: nearest template prediction
OS: overall survival
PAM: partition around medoids
PCA: Principal component analysis
ROC: receiver operating characteristic
scRNA-seq: single-cell RNA sequencing
SNV: single nucleotide variation
ssGSEA: single-sample gene set enrichment analysis
TCGA: The Cancer Genome Atlas
TME: tumor microenvironment
UCSC: University of California Santa Cruz
