# Supplementary figures and images for: Pan-cancer analysis of the transcriptional expression of histone acetylation enzymes in solid tumors defines a new classification scheme for gliomas
Source: Front Immunol. 2025 Jan 21;15:1523034. doi: 10.3389/fimmu.2024.1523034 (PMC11790639; doi:10.3389/fimmu.2024.1523034)

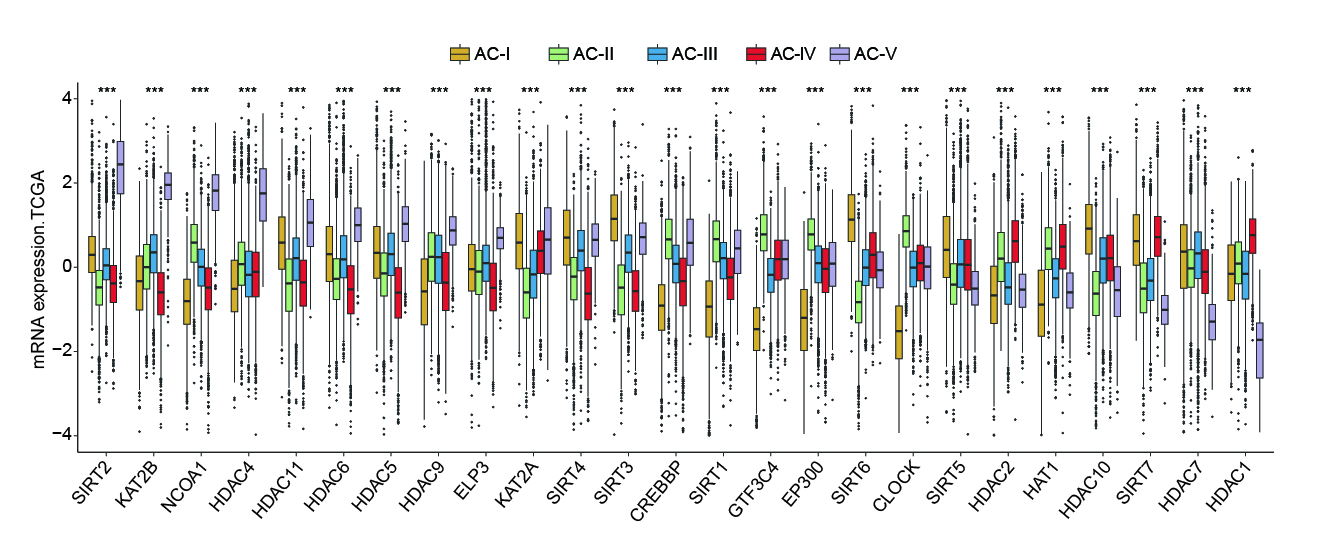

Supplement: Supplementary file 1 [file Image1.tif]

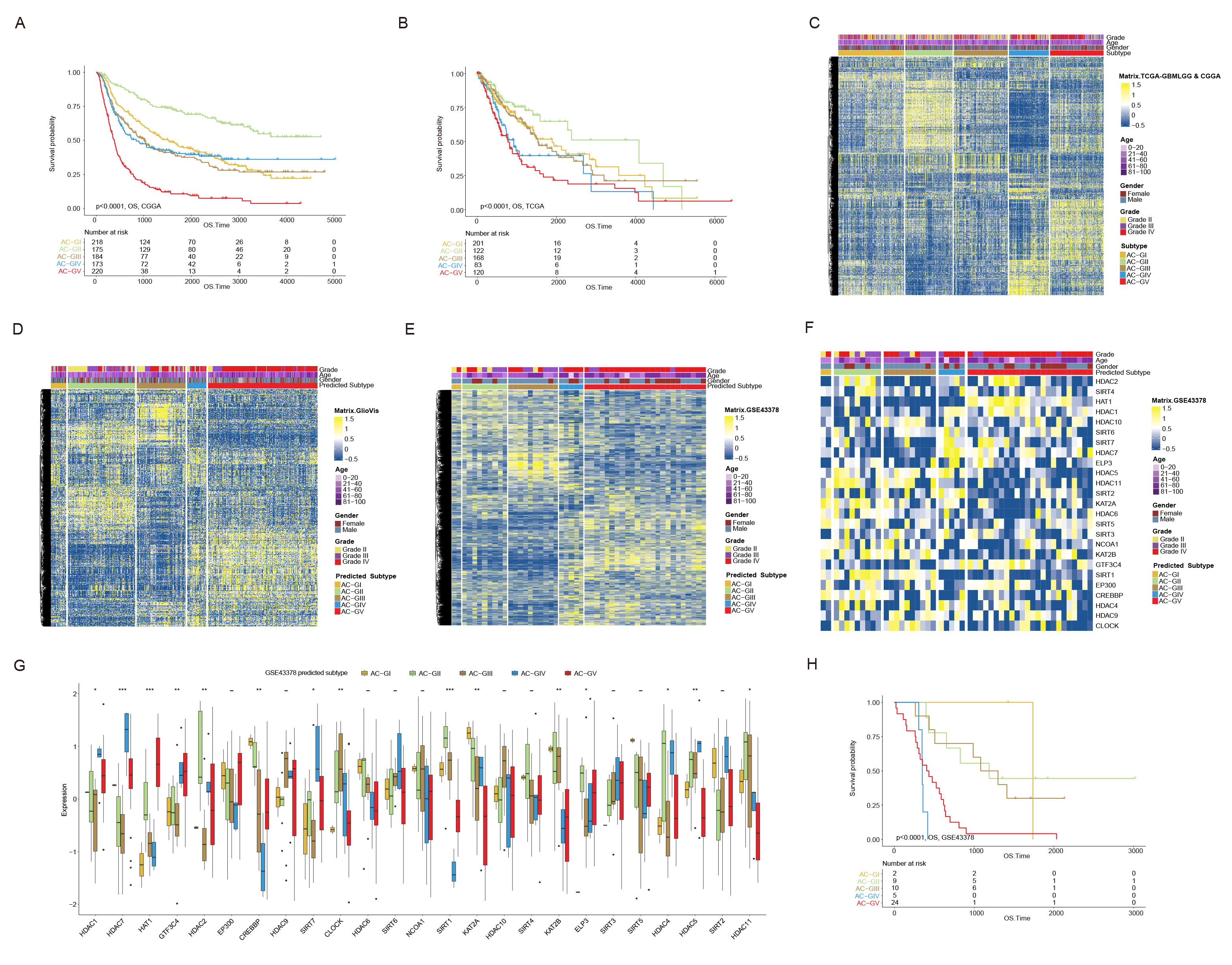

Supplement: Supplementary file 2 [file Image2.tif]

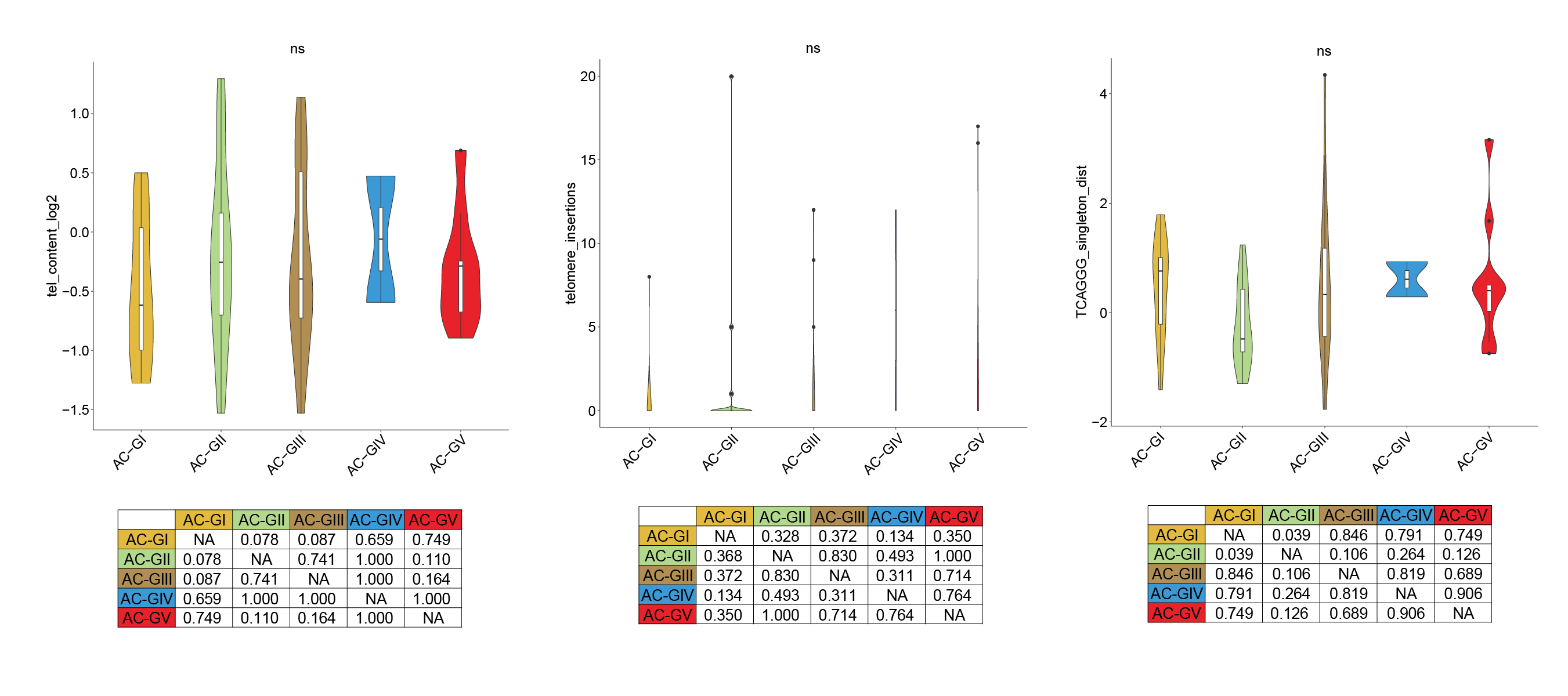

Supplement: Supplementary file 3 [file Image3.tif]

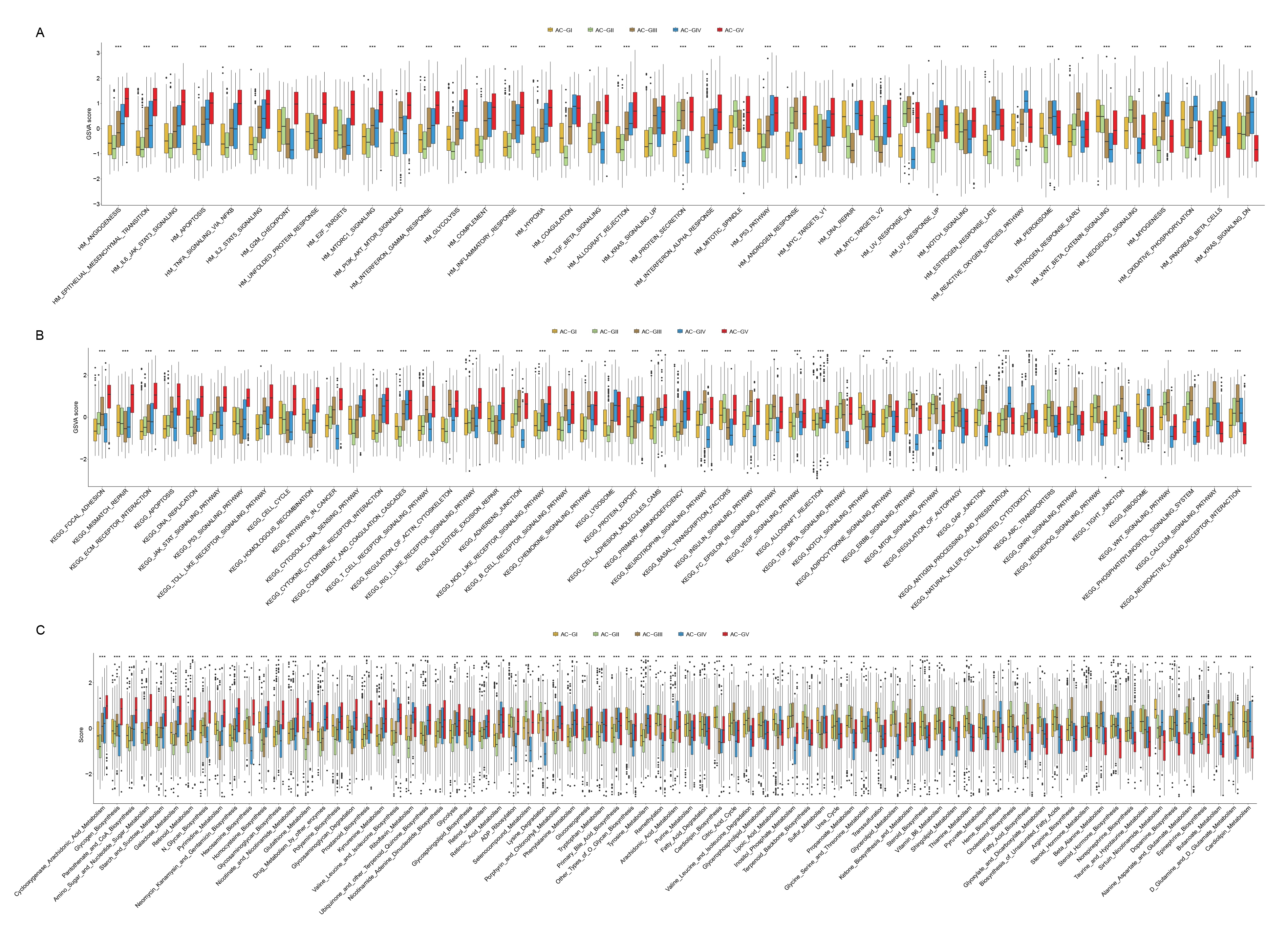

Supplement: Supplementary file 4 [file Image4.tif]

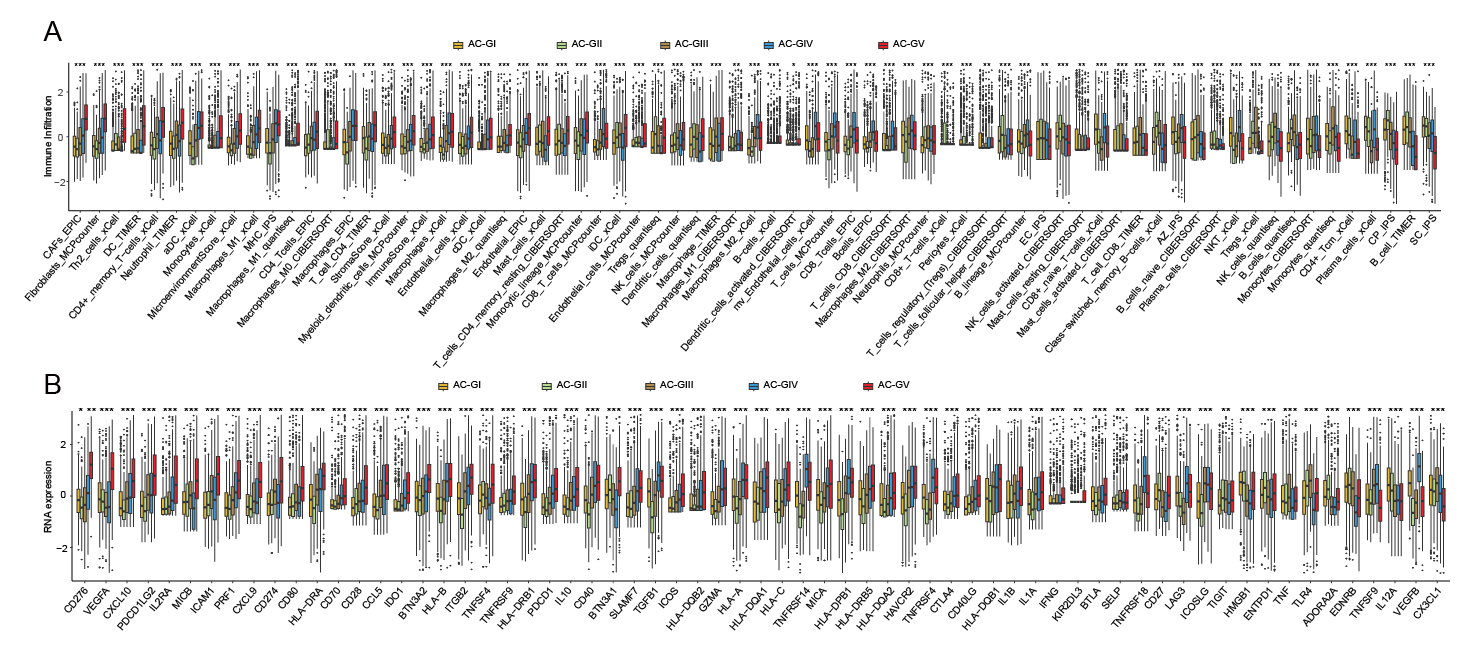

Supplement: Supplementary file 5 [file Image5.tif]

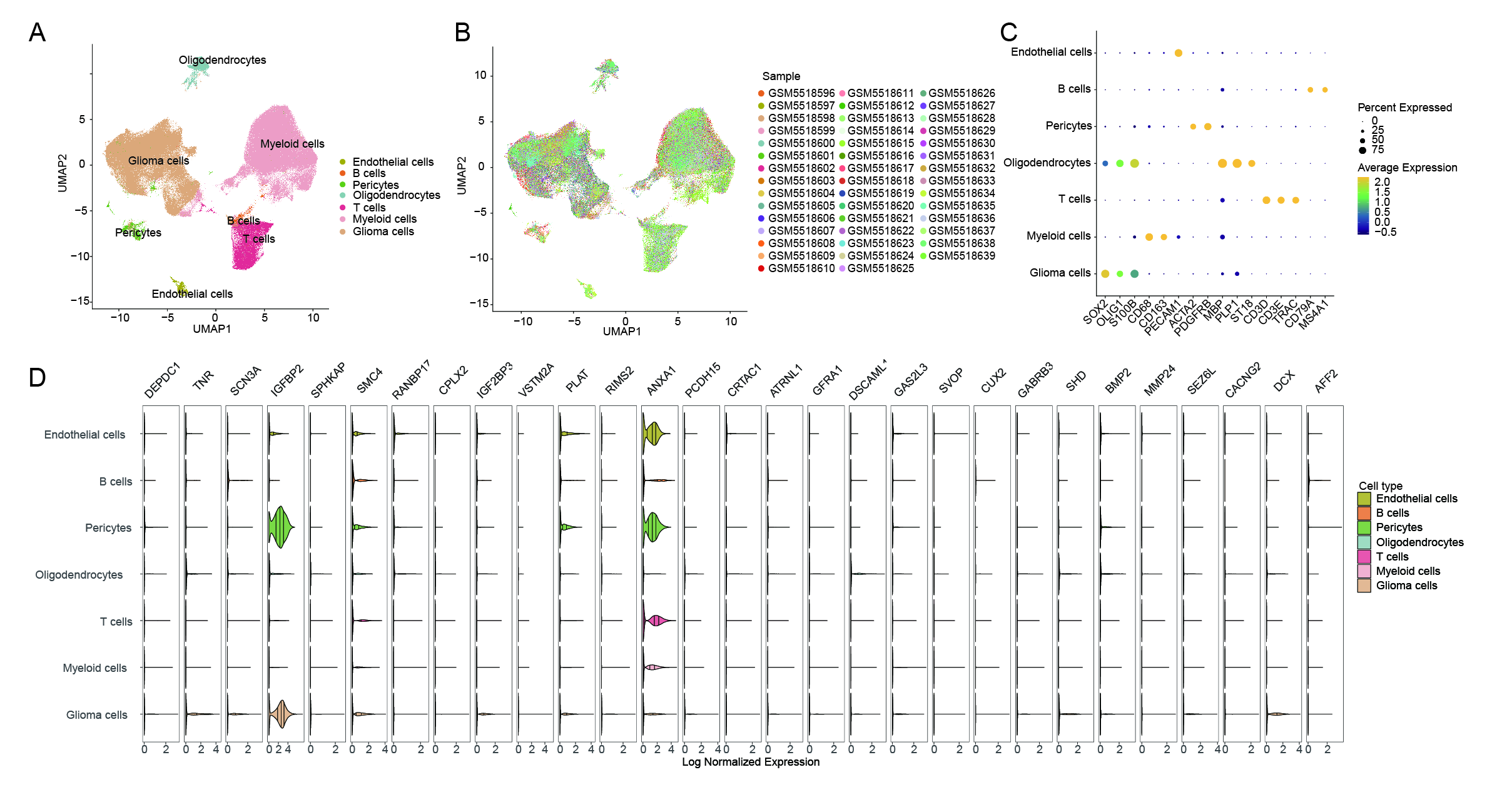

Supplement: Supplementary file 6 [file Image6.tif]

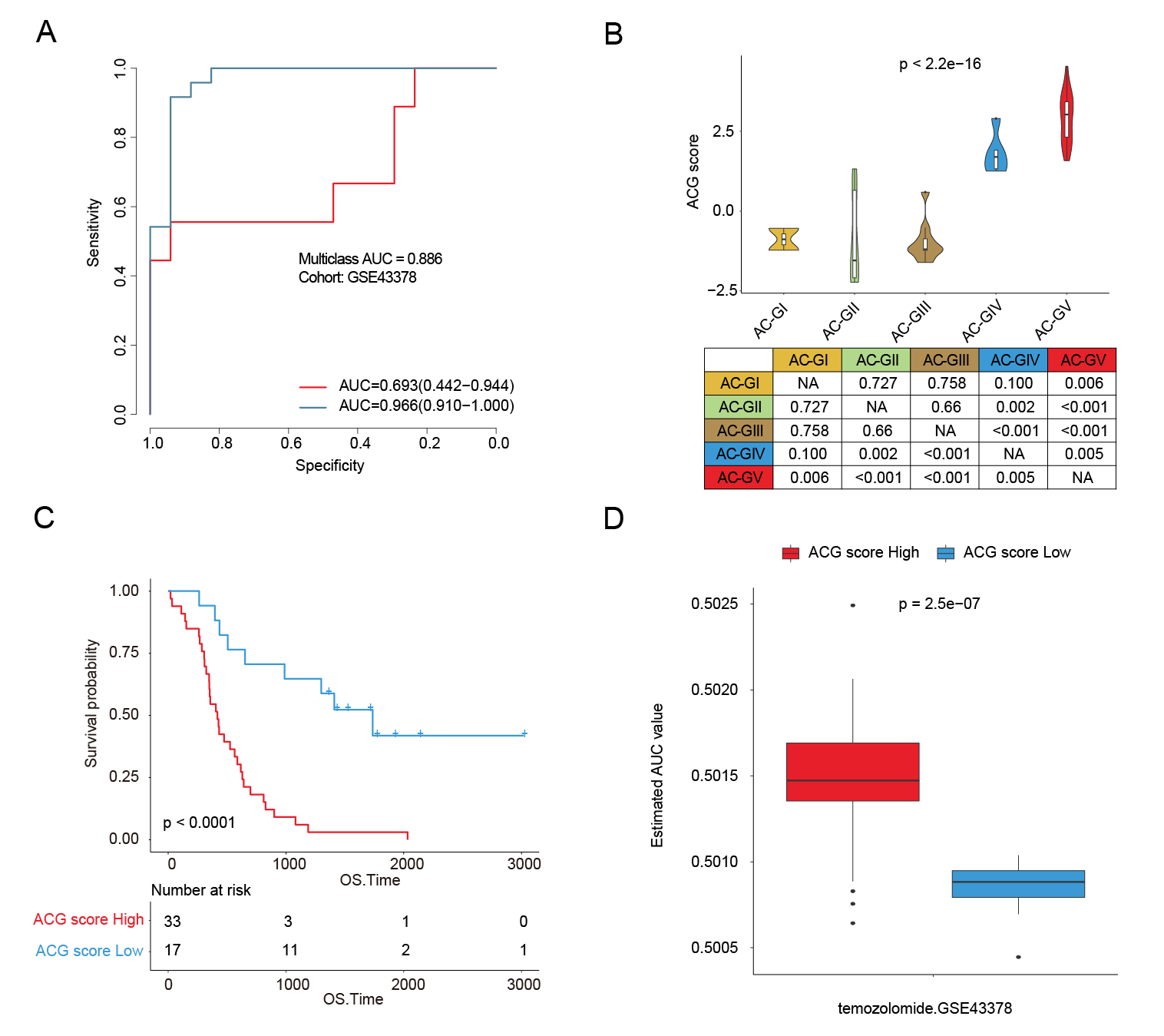

Supplement: Supplementary file 7 [file Image7.tif]

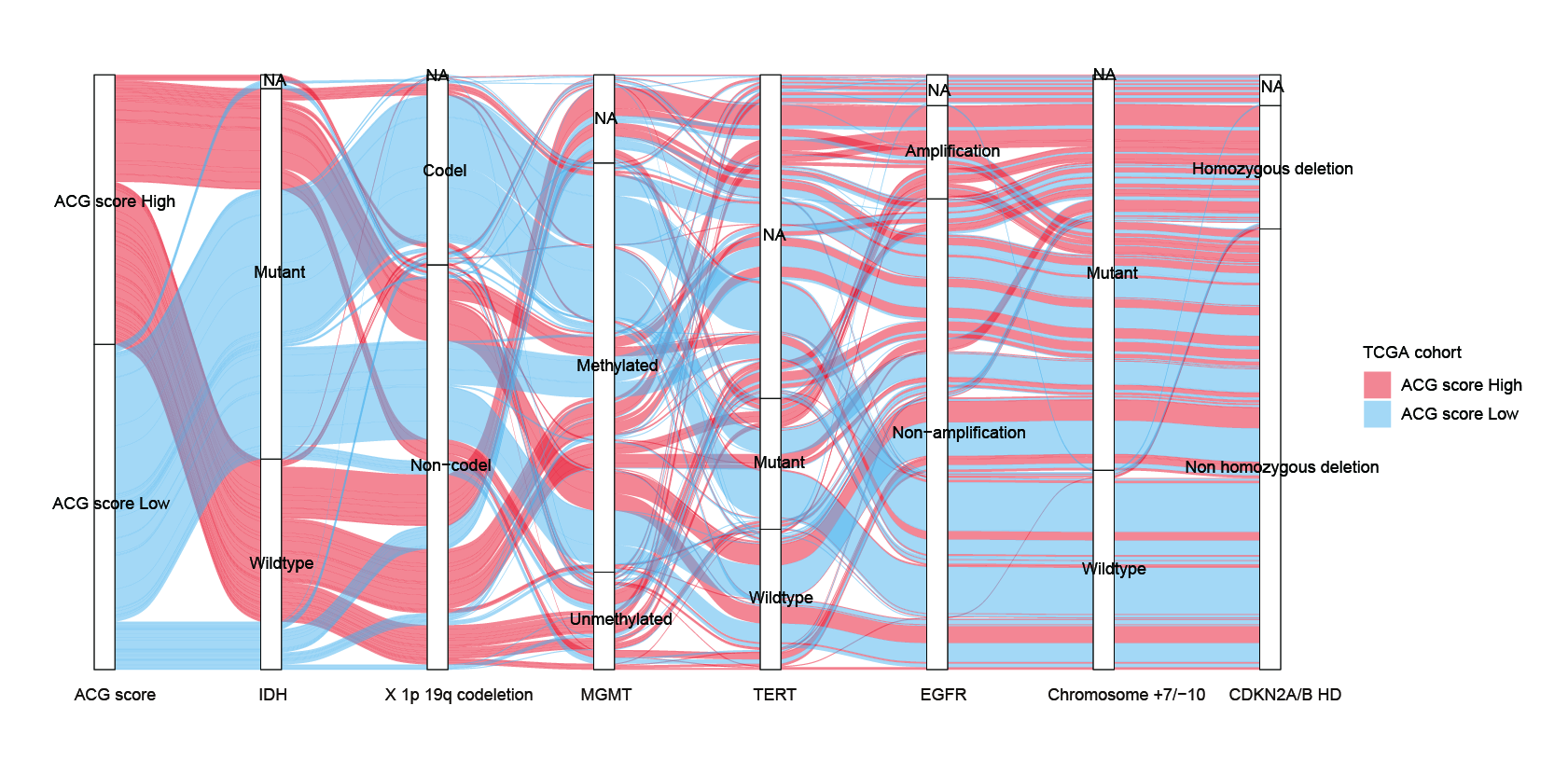

Supplement: Supplementary file 8 [file Image8.tif]
