# Supplementary material for: Pan-cancer analysis of the transcriptional expression of histone acetylation enzymes in solid tumors defines a new classification scheme for gliomas
Source: Front Immunol. 2025 Jan 21;15:1523034. doi: 10.3389/fimmu.2024.1523034 (PMC11790639; doi:10.3389/fimmu.2024.1523034)
Supplement: Supplementary file 9 [file Table1.docx]

**Supplementary Figure 1.** Expression patterns of ACRGs in AC subtypes. Box plots showed the mRNA expression of 25 ACRGs in different AC subtypes in the TCGA pan-cancer cohort. ***p < 0.001; ACRGs, acetylation regulator genes.

**Supplementary Figure 2.** (**A-C**) Heatmaps exhibited the expression of differently expressed genes among different ACG subtypes in the TCGA-GBMLGG & CGGA (**A**), GlioVis (**B**), and GSE43378 cohorts (**C**), respectively. (**D**) Heatmap depicted the expression pattern of the 25 ACRGs between different predicted ACG subtypes in the GSE43378 cohort. (**E**)Box plots showed the mRNA expression of 25 ACRGs in different predicted ACG subtypes in the GlioVis cohort. (**F-H**) Kaplan-Meier curves of overall survival (OS) according to different ACG subtypes in the CGGA (**F**), TCGA (**G**), and GSE43378 cohorts (**H**). *p < 0.05, **p < 0.01, ***p < 0.001

**Supplementary Figure 3.** Violin plots displayed the tel_content_log2 value (left), telomerase insertions (middle), and TCAGGG_singleton_dist value (right) among different ACG subtypes in TCGA-GBMLGG cohort. NA: not available.

**Supplementary Figure 4.** (**A-C**) Box plots depicted the Hallmark (**A**), KEGG biological pathways (**B**), and metabolism-related pathways (**C**) activation score. ***p < 0.001

**Supplementary Figure 5.** (**A-B**) Box plots depicted the tumor microenvironment cell infiltration level (**A**) and expression of different immune regulatory pathways genes (**B**) in different ACG subtypes. *p < 0.05, **p < 0.01, ***p < 0.001

**Supplementary Figure 6.** (**A-B**) UMAP projections of 214,366 aggregate single cells from 44 glioma samples showing the composition of different cell types in human gliomas. UMAP projections are shown by cluster assignment and by the patient. (**C**) Dot plots showing the average expression of known markers in indicated cell clusters. The dot size represents the percent of cells expressing the genes in each cluster. The expression intensity of markers is shown. (**D**)Violin plots showing the expression of 29 genes comprising “ACR score” model in tumor cells and immune cell clusters.

**Supplementary Figure 7.** (**A-B**) The receiver operating characteristics curve (**A**) and violin plots (**B**) showed the ability of ACG score to distinguish predicted AC-GV (blue) and predicted AC-GII subtype (red) from other ACG subtypes in the GSE43378 cohort. (**C**) Kaplan-Meier curves of OS in the GSE43378 cohort. (**D**) Box plots exhibited predicted area under curve values of temozolomide among different ACG score groups based on CTRP analysis in the GSE43378 cohorts. NA: not available.

**Supplementary Figure 8.** Sankey diagram of high and low ACG groups with different molecular subtypes in the TCGA-GBMLGG cohort.
